# ROCK1/2 signaling contributes to corticosteroid-refractory acute graft-versus-host disease

**DOI:** 10.1038/s41467-024-44703-7

**Published:** 2024-01-10

**Authors:** Kristina Maas-Bauer, Anna-Verena Stell, Kai-Li Yan, Enrique de Vega, Janaki Manoja Vinnakota, Susanne Unger, Nicolas Núñez, Johana Norona, Nana Talvard-Balland, Stefanie Koßmann, Carsten Schwan, Cornelius Miething, Uta S. Martens, Khalid Shoumariyeh, Rosa P. Nestor, Sandra Duquesne, Kathrin Hanke, Michal Rackiewicz, Zehan Hu, Nadia El Khawanky, Sanaz Taromi, Hana Andrlova, Hemin Faraidun, Stefanie Walter, Dietmar Pfeifer, Marie Follo, Johannes Waldschmidt, Wolfgang Melchinger, Michael Rassner, Claudia Wehr, Annette Schmitt-Graeff, Sebastian Halbach, James Liao, Georg Häcker, Tilman Brummer, Joern Dengjel, Geoffroy Andrieux, Robert Grosse, Sonia Tugues, Bruce R. Blazar, Burkhard Becher, Melanie Boerries, Robert Zeiser

**Affiliations:** 1 Department of Medicine I, Medical Center - University of Freiburg, Faculty of Medicine, University of Freiburg, Freiburg, Germany; 2 Faculty of Biology, University of Freiburg, Freiburg, Germany; 3 Institute of Experimental Immunology, University of Zurich, Zurich, Switzerland; 4 Institute of Experimental and Clinical Pharmacology and Toxicology, Medical Faculty, University of Freiburg, Freiburg, Germany; 5 German Cancer Consortium (DKTK), Partner Site Freiburg, a partnership between German Cancer Research Center (DKFZ) and Medical Center - University of Freiburg, Freiburg, Germany; 6 Department of Biology, University of Fribourg, Fribourg, Switzerland; 7 Department of Dermatology, Medical Center, University of Freiburg, Freiburg, Germany; 8 Institute of Pathology, University Hospital Freiburg, Freiburg, Germany; 9 IMMZ, University of Freiburg, Faculty of Medicine, Freiburg, Germany; 10 Department of Medicine, University of Arizona, Tucson, USA; 11 IMMH, University Hospital Freiburg, Faculty of Medicine, Freiburg, Germany; 12 Signaling Research Centres BIOSS and CIBSS - Centre for Integrative Biological Signaling Studies, University of Freiburg, Freiburg, Germany; 13 Institute of Medical Bioinformatics and Systems Medicine, Medical Center – University of Freiburg, Faculty of Medicine, University of Freiburg, Freiburg, Germany; 14 CIBSS-Centre for Integrative Biological Signalling Studies, University of Freiburg, Freiburg, Germany; 15 Department of Pediatrics, Division of Blood & Marrow Transplant & Cellular Therapy, University of Minnesota, Minneapolis, MN USA

**Keywords:** Translational research, Graft-versus-host disease

## Abstract

Patients with corticosteroid-refractory acute graft-versus-host disease (aGVHD) have a low one-year survival rate. Identification and validation of novel targetable kinases in patients who experience corticosteroid-refractory-aGVHD may help improve outcomes. Kinase-specific proteomics of leukocytes from patients with corticosteroid-refractory-GVHD identified rho kinase type 1 (ROCK1) as the most significantly upregulated kinase. ROCK1/2 inhibition improved survival and histological GVHD severity in mice and was synergistic with JAK1/2 inhibition, without compromising graft-versus-leukemia-effects. ROCK1/2-inhibition in macrophages or dendritic cells prior to transfer reduced GVHD severity. Mechanistically, ROCK1/2 inhibition or ROCK1 knockdown interfered with CD80, CD86, MHC-II expression and IL-6, IL-1β, iNOS and TNF production in myeloid cells. This was accompanied by impaired T cell activation by dendritic cells and inhibition of cytoskeletal rearrangements, thereby reducing macrophage and DC migration. NF-κB signaling was reduced in myeloid cells following ROCK1/2 inhibition. In conclusion, ROCK1/2 inhibition interferes with immune activation at multiple levels and reduces acute GVHD while maintaining GVL-effects, including in corticosteroid-refractory settings.

## Abstract (web summary)

Steroid-refractory acute graft-versus-host disease (aGVHD) is associated with a low one-year survival rate. Here, the authors show that ROCK1 is upregulated in leukocytes from patients with steroid-refractory aGVHD and that ROCK1/2 inhibition reduces the severity of aGVHD in mice by interfering with activation of multiple immune cell types.

## Introduction

Acute graft-versus-host disease (aGVHD) is a major complication after allogenic hematopoietic cell transplantation (allo-HCT). Overall, 30–60% of patients develop grade II-IV aGVHD, 14% grade III-IV aGVHD^1–3^ and 30–40% chronic GVHD (cGVHD)^4^. aGVHD pathophysiology is based on the activation of donor T cells that expand and attack the tissues of the recipient. In recent years, several groups including our own have shown that also innate immune cells can contribute to aGVHD including neutrophil granulocytes (neutrophils)^5,6^ and inflammatory monocytes^7,8^, which are activated by pathogen-associated molecular patterns and damage-associated molecular patterns^9,10^. The first line treatment of aGVHD are corticosteroids with a response rate of 40–60%. Patients with corticosteroid-refractory (SR) aGVHD (SR-aGVHD) have a dismal prognosis with a reported one-year survival rate that ranges between 10 and 38%^11^. Most second line therapeutic regimens such as calcineurin inhibitors, antimetabolites (methotrexate and mycophenolate)^12^, or anti-thymocyte globulin^13,14^ target the alloreactive T cell activation and expansion. Since these T cell-targeted therapies are not overall successful, it is conceivable that besides T cells, other immune cells maintain the inflammatory local milieu that promotes steroid-refractoriness. Rho-associated coiled-coil-containing protein kinase (ROCK) 1 and 2 are two isoforms that share 92% homology in their kinase domain^15^ and are activated when bound to the GTP-bound form of Rho, thereby regulating the phosphorylation of downstream targets, including LIM kinase 1 and 2^16^ and MYPT1^17^. ROCKs are engaged in various cellular activities including cell shape, migration capabilities, proliferation, and apoptosis^18–20^. In the last decades, ROCK1 and 2 have been identified as important molecules in tumorigenesis^21,22^ cardiovascular diseases^23^ and autoimmunity in mice^24^, moreover T-cell receptor (TCR) signaling and the acquisition of the appropriate T cell effector program was shown to be dependent on ROCK1^25^. In line with these findings, ROCK2 has been demonstrated to downregulate pro-inflammatory cytokines, such as Il-17 and IL-21 via a STAT3-dependent mechanism^26,27^ and the ROCK2-inhibitor belumosudil has recently been approved by the FDA for the treatment of SR-cGVHD^28^. In this study, using an unbiased approach, we report that ROCK1 is highly upregulated in myeloid cells of patients with SR-aGVHD. We also test the hypothesis that ROCK1/2 inhibition reduces myeloid cell activation and thereby also aGVHD severity in a murine GVHD model.

## Results

### ROCK1/2 are found in myeloid cells during SR-aGVHD

**Fig. 1:**
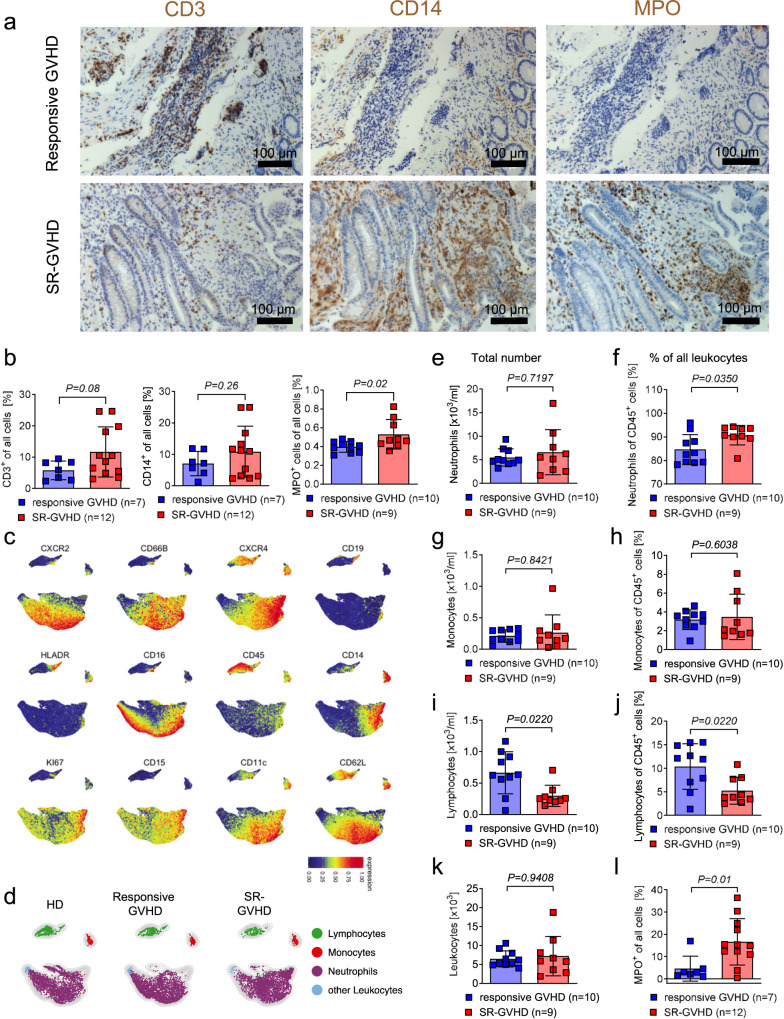
Neutrophils and monocytes are increased in aGVHD lesions and blood of SR-aGVHD patients. **a** Representative immunohistochemistry staining (brown) for CD3, CD14, and myeloperoxidase (MPO) in intestinal tissue biopsies derived from patients with SR-aGVHD or patients with steroid responsive aGVHD, Scale bar: 100 µm. **b** Quantification of CD3^+^, CD14^+^ or MPO^+^ cells in intestinal tissue biopsies derived from patients with SR-aGVHD (*n* = 12 for CD3^+^ and CD14^+^, *n* = 9 for MPO^+^) versus responsive GVHD (*n* = 7 for CD3^+^ and CD14^+^, *n* = 10 for MPO^+^). Each dot represents one patient. Percentage represents positive cells / all cells (36 high power fields analyzed per sample) are shown. Plots show mean +/− SD and *P*-value was calculated by two-tailed unpaired student *t*-test. **c** Expression intensity of indicated markers on stochastically selected leukocytes (defined as single/CD45^+^) from healthy donors (HD, *n* = 12), steroid-responsive aGVHD patients (*n* = 10) and SR-aGVHD patients (*n* = 9) visualized by UMAP. **d** FlowSOM clustered leukocyte subsets are overlaid on the UMAP plot for each condition separately. Leukocytes were derived from healthy donors (HD, *n* = 12), steroid-responsive aGVHD patients (*n* = 10) and SR-aGVHD patients (*n* = 9) visualized by UMAP. **e**–**j** Frequency and absolute counts of neutrophils, monocytes, and lymphocytes from steroid-responsive GVHD (*n* = 10) and SR-aGVHD patients (*n* = 9). Each dot represents one patient. Plots show mean +/− SD and *P*-value was calculated by Mann–Whitney test. **k** Absolute counts of leukocytes from steroid-responsive GVHD (*n* = 10) and SR-aGVHD patients (*n* = 9). Each dot represents one patient, plots show mean +/− SD and *P*-value was calculated by Mann–Whitney test. **l** The scatter plot shows MPO^+^ cells relative to all cells in percent derived from the peripheral blood of patients with steroid-responsive aGVHD (*n* = 7) or SR-aGVHD (*n* = 12). Each dot represents one patient. Plots show mean +/− SD and *P*-value was calculated two-tailed unpaired student *t*-test.

To characterize the immune cell types found in aGVHD lesions of patients who had failed corticosteroids, we analyzed human intestinal biopsies. We observed that myeloperoxidase (MPO) positive cells increased in SR-aGVHD lesions as compared to biopsies of patients post allo-HCT that had steroid-responsive aGVHD (Fig. 1a, b). CD14^+^ monocytes and T cells (CD3^+^) were also detectable in SR-aGVHD (Fig. 1a, b). A high dimensional flow-cytometry-based analysis performed on leukocytes derived from the peripheral blood of healthy donors or patients with responsive aGVHD or SR-aGVHD revealed that SR-aGVHD patients exhibited a reduced frequency and absolute numbers of lymphocytes leading to relatively increased percentages of neutrophils (Fig. 1c–k). Conversely, there was no difference in monocyte frequencies or absolute numbers in patients with responsive aGVHD compared to SR-aGVHD (Fig. 1c–k). Furthermore, we found CD45^+^ cells from SR-aGVHD patients to express higher levels of myeloperoxidase in comparison to those from aGVHD patients that responded to steroids (Fig. 1l).

**Fig. 2:**
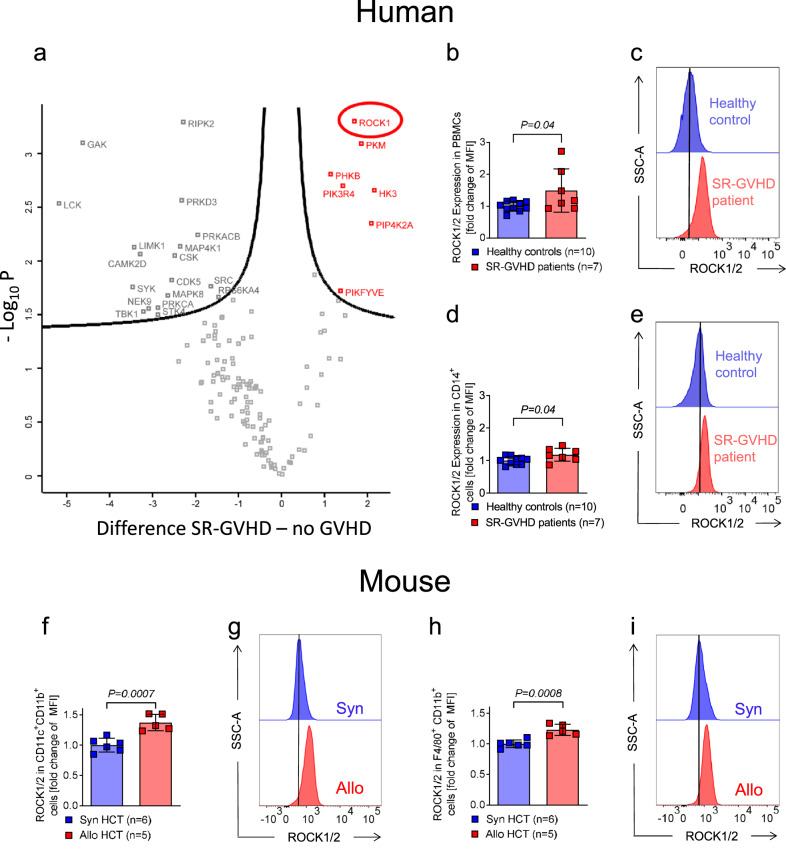
ROCK1/2 is increased in patients with aGVHD and in mice after allogenic BMT. **a** The volcano plot shows significant protein abundance differences of activated kinases isolated from patients without aGVHD (*n* = 6) and patients with SR-aGVHD (*n* = 6). Kinases were enriched by kinase-bead based selection as reported by others^41^. The graph shows in red the kinases that were significantly more abundant in patients with SR-aGVHD (*t*-test, permutation-based FDR < 0.05). **b** The scatter plot shows the quantification (fold change of MFI) of ROCK1/2 expression in PBMCs of healthy controls versus SR-aGVHD patient samples. Each data point represents an individual patient and values were normalized to the mean of the control group. The plot show mean +/− SD and the *P*-value was calculated using a two-tailed unpaired student *t*-test. **c** Representative histograms of ROCK1/2 expression in PBMCs of healthy controls and SR-aGVHD patients as indicated. **d** The scatter plot shows the quantification (fold change of MFI) of ROCK1/2 expression in CD14^+^ cells of healthy controls versus SR-aGVHD patient samples. Each data point represents an individual patient and values were normalized to the mean of the control group. The plot show mean +/− SD and the *P*-value was calculated using a two-tailed unpaired student *t*-test. **e** Representative histograms of ROCK1/2 expression in CD14^+^ cells of healthy controls and GVHD patients as indicated. **f** The scatter plot shows the quantification (fold change of MFI) of ROCK1/2 expression in CD11c^+^CD11b^+^ DCs isolated from the spleen of mice on day 14 after syngeneic HCT or allogeneic HCT as indicated. The experiment was performed three times, each data point represents one mouse and values were normalized to the mean of the control group. The plot show mean +/− SD and the *P*-value was calculated using a two-tailed unpaired student *t*-test. **g** Representative histograms of ROCK1/2 expression in CD11c^+^CD11b^+^ DCs. **h** The scatter plot shows the quantification (fold change of MFI) of ROCK1/2 expression in CD11b^+^ F4/80^+^ macrophages isolated from the spleen of mice on day 14 after syngeneic HCT or allogeneic HCT as indicated. The experiment was performed two times, each data point represents one mouse and values were normalized to the mean of the control group. The plot show mean +/− SD and the *P*-value was calculated using a two-tailed unpaired student *t*-test. **i** Representative histograms of ROCK1/2 expression in CD11b^+^ F4/80^+^ macrophages.

Based on the high levels of MPO positive cells and neutrophils found in the blood and in lesions from SR-aGVHD patients, we next isolated leukocytes from peripheral blood and submitted them to protein isolation and Kinet-1 bead-based enrichment of activated and hence ATP analogue binding kinases as reported by others^29^. Mass-spectrometry-based proteomics analysis revealed seven highly upregulated kinases in SR-aGVHD compared to patients post allo-HCT that did not have aGVHD (Fig. 2a). Five identified kinases were involved in metabolic processes (PKM, PHKB, PIK3R4, HK3, and PIP4K2A) and difficult to target pharmacologically because of potential side effects. ROCK1 was most abundantly upregulated and is a targetable kinase with a selective inhibitor that is commercially available^30^. We therefore chose ROCK1 for further studies and validated the proteomics data with flow-cytometry. Using an antibody that binds both ROCK1 and ROCK2, we found that these molecules were more highly expressed in SR-aGVHD patients compared to healthy individuals, in peripheral blood mononuclear cells (PBMC) (Fig. 2b, c) and in CD14^+^ cells (Fig. 2d, e).

To validate our findings in humans also in mice, we next studied ROCK1/2 in dendritic cells (DC) and macrophages isolated from mice undergoing allogeneic-HCT compared to syngeneic-HCT. We observed that ROCK1/2 was upregulated in DCs and macrophages isolated from mice undergoing allogeneic-HCT compared to syngeneic-HCT (Fig. 2f–i). These findings indicate that the connection between aGVHD and ROCK1/2 expression seen in patients also exists in mice. The association of aGVHD and the abundance of ROCK1/2 expressing leukocytes prompted us to further investigate the function of ROCK1/2 in the development of aGVHD to evaluate if a pathway intervention has therapeutic potential.

### ROCK1/2-inhibition reduces aGVHD severity and immune activation at multiple levels in myeloid cells

**Fig. 3:**
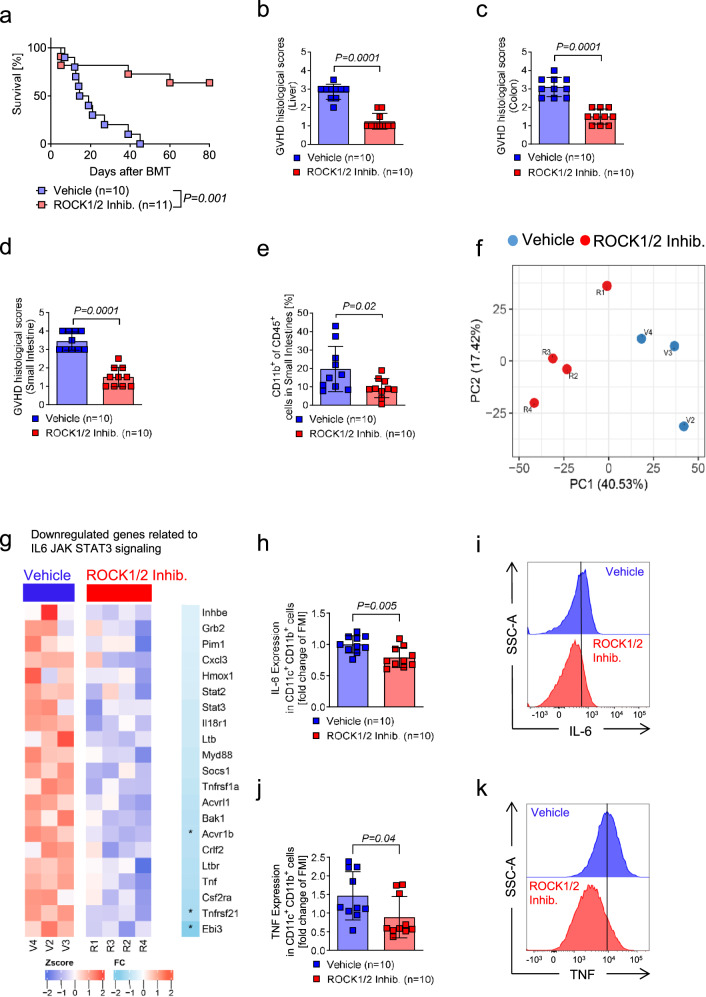
ROCK1/2-inhibition reduces aGVHD in mice and causes downregulation of genes related to IL6 JAK STAT3 signaling in CD11b^+^ cells. **a**–**k** Mice underwent allo-HCT to induce aGVHD. Mice in the ROCK1/2-inhibitor group were treated intraperitoneal with 8 mg/kg ROCK1/2-inhibitor (dissolved in 100 µl PBS) from day 4 to day 13, mice in the vehicle group were treated intraperitoneal with an equal volume PBS. **a** Survival of mice was plotted by using the Kaplan–Meier method and compared by using a log-rank (Mantel Cox) test. Depicted are 10 mice in the vehicle group and 11 mice in the ROCK1/2-inhibitor group. The experiment was performed twice. **b**–**d** Histological aGVHD scoring of liver (**b**), colon (**c**) and small intestine (**d**) of mice on day 14 after allo-HCT that received either ROCK1/2-inhibitor or vehicle treatment. Depicted are 10 mice in each group. The plots show mean +/− SD and the *P*-value was calculated using a two-tailed unpaired student *t*-test. **e** The scatter plot shows the percentage of CD11b^+^ cells of all CD45^+^ cells in the lamina propria of small intestine on day 14 after allo-HCT, which equals 10 days of ROCK1/2-inhibitor or vehicle treatment. Depicted are 10 mice each group. The experiment was performed twice. The plot show mean +/− SD and the *P*-value was calculated using a two-tailed unpaired student *t*-test. **f** Principal Component (PC) Analysis based on gene expression, determined by mRNA-microarray of CD45^+^CD11b^+^ cells that were FACS-isolated from the spleen of mice on day 14 after allo-HCT, which equals 10 days of ROCK1/2-Inhibitor or vehicle treatment. Depicted are four mice in the ROCK1/2-inhibitor group (red) and three mice in the vehicle group (blue). **g** The heatmap shows downregulated genes involved in IL6_JAK_STAT3_signaling. Color scale represents the row-wise z-score intensity. * indicates significant changes in gene expression. **h** The scatter plot shows the MFI of *Il-6* expression in CD11c^+^CD11b^+^ DCs isolated from the spleen on day 14 after allo-HCT. The experiment was performed three times, each data point represents one mouse and values were normalized to the mean of the control group. The plot show mean +/− SD and the *P*-value was calculated using a two-tailed unpaired student *t*-test. **i** Representative histograms of *Il-6* expression in CD11c^+^CD11b^+^ DCs isolated from the spleen on day 14 after allo-HCT. **j** The scatter plot shows the MFI of *Tnf* expression in CD11c^+^CD11b^+^ DCs isolated from the spleen on day 14 after allo-HCT. The experiment was performed three times and each data point represents one mouse. The plot show mean +/− SD and the *P*-value was calculated using an two-tailed unpaired student *t*-test. **k** Representative histograms of *Tnf* expression in CD11c^+^CD11b^+^ DCs isolated from the spleen on day 14 after allo-HCT.

To understand if ROCK1/2-inhibition has therapeutic activity against aGVHD we treated recipient mice (C57BL/6 into BALB/c) with the ROCK1/2-inhibitor Y-27632. To date, no fully ROCK1 isoform-specific inhibitor has been generated because the ROCK1 and ROCK2 isoforms exhibit 90% sequence homology in their ATP binding sites^15,31^. Y-27632 inhibits the kinase activity of both isoforms with the following dissociation constant of the enzyme-inhibitor complex (Ki): ROCK1 (Ki = 140/220 nM) and ROCK2 (Ki = 300 nM)^32^. We found that ROCK1/2-inhibition with Y-27632 improved survival compared to vehicle (Fig. 3a) and reduced aGVHD histopathology scores (Fig. 3b–d). The scores were determined on the basis of crypt apoptosis (0-4) and inflammation (0-4) in the small and large intestines, and liver was assessed for bile duct injury (manifest by nuclear hyperchromasia, nuclear crowding, infiltrating lymphocytes, and cytoplasmic vacuolation) and inflammation (infiltration with lymphocytes, neutrophils and eosinophils). Disease was scored between 0 and 4 based on the number of involved tracts and the severity of disease in each tract^33^. ROCK1/2-inhibition did not interfere with engraftment as a 100% donor chimerism was seen on day 30 after allo-HCT in vehicle and ROCK1/2-inhibitor treated mice (Supplementary Fig. 1a–d). Moreover, ROCK1/2-inhibition led to a reduction of CD11b^+^ cells within the CD45^+^ cells in the small intestines (Fig. 3e). Gene expression analysis of CD11b^+^ splenocytes isolated on day 14 after allo-HCT revealed a separation of the cluster of vehicle-treated mice compared to the cluster of ROCK1/2-inhibitor treated mice (Fig. 3f). The analysis also revealed a reduced expression of genes related to *IL-6* and *JAK-STAT3* signaling in the ROCK1/2-inhibitor group (Fig. 3g), which are pro-inflammatory signaling events with a known connection to aGVHD in mice^34^ and patients^35^. To validate these results on the protein level we used flow-cytometry and found a decrease of the pro-inflammatory cytokines IL-6 and TNF in CD11c^+^CD11b^+^ cells isolated from the spleens of mice treated with ROCK1/2-inhibitor compared to mice treated with vehicle (Fig. 3h–k). Both IL-6 and TNF are cytokines with a known role in aGVHD^36^.

### ROCK1/2-inhibition reduces stimulatory activity, co-stimulatory molecule and pro-inflammatory cytokine expression and migratory activity of DCs

**Fig. 4:**
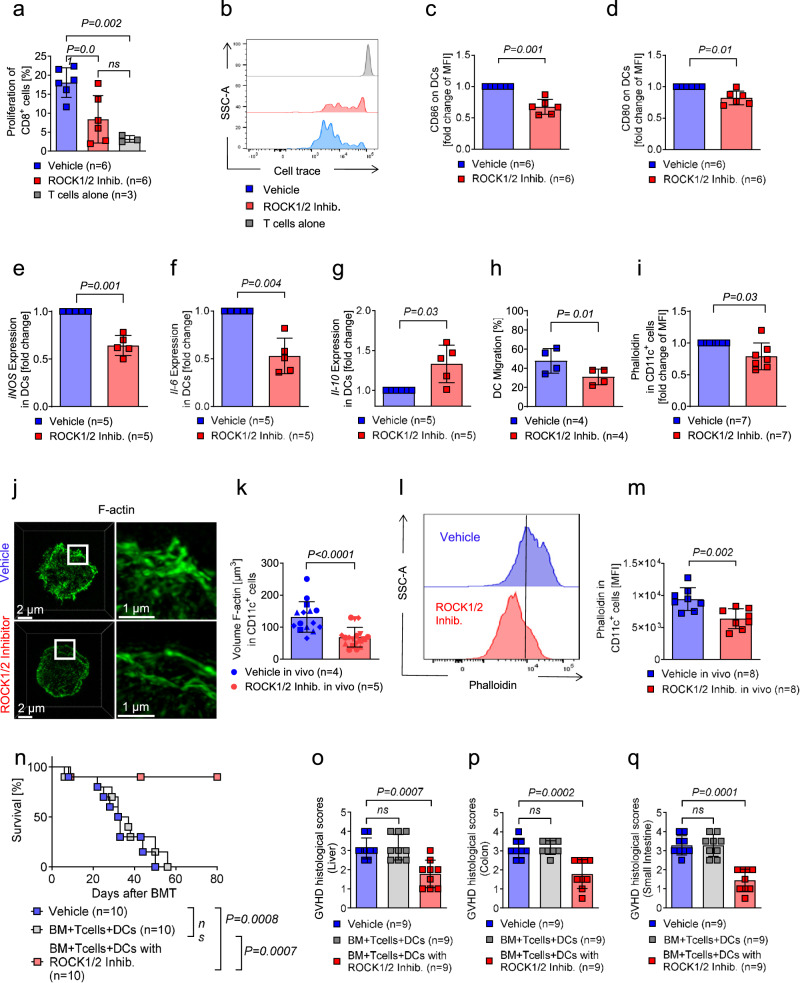
ROCK1/2-inhibition reduces stimulatory activity, co-stimulatory molecule expression, *Il-6*, *iNOS*, and migratory activity of DCs. **a**–**d** Coculture of T cells and allogeneic BM-derived DCs was performed for 72 hours. DCs were activated with LPS for 24 h and when indicated thereafter treated with 30 µg/ml ROCK1/2-inhibitor for two hours prior to the coculture. **a** The scatter plot shows the proliferation of CD8^+^ T cells in response to coculture with allogeneic DCs. The experiment was performed three times, each point represents one mouse. The plot shows mean +/− SD and the *P*-value was calculated using a two-way ANOVA (Dunnett’s multiple comparison). **b** Representative histogram showing the proliferation of CD8^+^ T cells in response to coculture with allogeneic DCs. **c,** **d** The scatter plots show the fold change in the mean fluorescence intensity (MFI) of the surface marker CD86 (**c**) and CD80 (**d**) on CD11c^+^ cells incubated in a coculture with T cells. The experiment was performed three times, each point represents one mouse. The plots show mean +/− SD and the *P*-value was calculated using a two-tailed paired student *t*-test. **e**–**g** The scatter plots show the fold change of *iNOS* mRNA (**e**), *Il*-6 mRNA (**f**) and *Il-10* (**g**) mRNA isolated from CD11c^+^ cells. BM-derived DCs were treated with LPS for two hours and exposed to ROCK1/2-Inhibitor or vehicle for two hours. The experiment was performed three times, each point represents a mouse, values of the experimental groups were normalized to vehicle control. The plots show mean +/− SD and the *P*-value was calculated using a two-tailed paired student *t*-test. **h** The scatter plot shows the migration of CD11c^+^ cells. BM-derived DCs were treated with LPS for two hours and exposed to ROCK1/2-inhibitor for two hours. Thereafter, they were incubated in a transwell chamber for five hours with a CXCL12 gradient and migration was assessed using FACS. The experiment was performed three times, each point represents one mouse. The plot shows mean +/− SD and the *P*-value was calculated using a two-tailed paired student *t*-test. **i** The scatter plot shows the fold change of MFI for phalloidin in CD11c^+^ cells. BM-derived DCs were treated with LPS for two hours and exposed to ROCK1/2-inhibitor for two hours. The experiment was performed three times, each point represents one mouse, values of the experimental group were normalized to vehicle control. The plot shows mean +/− SD and the *P*-value was calculated using a two-tailed paired student *t*-test. **j** The representative images show staining for phalloidin (green) in CD11c^+^ cells isolated from mice that had undergone allo-HCT. Mice were treated intraperitoneal with vehicle or ROCK1/2- inhibitor (8 mg/kg in 100 µl PBS) from day 4 to day 13. Images (z-stacks) were acquired by structured illumination microscopy (SIM). Superresolution images were processed with Zen Black software and visualized in 3D with Imaris software (maximum intensity projection). All scale bars are 2 µm in overviews and 1 µm in magnifications. **k** The scatter plot shows the Volume [µm^3^] of F-actin in CD11c^+^ cells isolated from mice that had undergone allo-HCT. Mice were treated intraperitoneal with vehicle or 8 mg/Kg ROCK1/2-inhibitor from day 4 to day 13. Cells were isolated from mice of two independent experiments and analyzed. Individual symbols represent measurements of cells isolated from the same animal. The plot shows mean +/− SD and *P*-value was calculated using an two-tailed unpaired student *t*-test. **l** Representative histogram showing phalloidin staining of F-actin in CD11c^+^ cells isolated from mice that had undergone allo-HCT as described in (**k**). **m** The scatter plot shows the MFI for phalloidin staining of F-actin in CD11c^+^ cells isolated from mice that had undergone allo-HCT. Mice were treated intraperitoneal with vehicle or ROCK1/2-inhibitor (8 mg/kg in 100 µl PBS) from day 4 to day 13. The experiment was performed twice, each point represents one mouse. The plot shows mean +/− SD and the *P*-value was calculated using an two-tailed unpaired student *t*-test. **n** Survival of mice undergoing allo-HCT to induce aGVHD. On the same day, BM-derived DCs were incubated with ROCK1/2-inhibitor or PBS for four hours, thereafter 1 × 10^6^ donor DCs were injected together with the graft. Depicted are 10 mice in each group from two individual experiments. Survival of mice was plotted by using the Kaplan-Meier method and compared by using a log-rank (Mantel Cox) test. **o**–**q** Histological aGVHD scoring of liver (**o**), colon (**p**) and small intestine (**q**) of mice on day 14 after allo-HCT as described in (**n**). The plots show mean +/− SD and the *P*-value was calculated using a two-way ANOVA (Dunnett’s multiple comparison).

A central event in the pathogenesis of aGVHD is the activation of CD8^+^ effector T cells by DCs. To test if ROCK1/2-inhibition affects the stimulatory activity of DCs, we exposed DCs to Y-27362 for two hours, then washed the DCs and used them as stimulator for allogeneic T cells. The percentage of proliferating CD8^+^ cells was reduced when DCs had been exposed to ROCK1/2-inhibitor compared to DCs exposed to vehicle (Fig. 4a, b). Consistent with reduced stimulatory activity of the DCs, the expression of CD80 and CD86 was reduced on DCs exposed to ROCK1/2-inhibitor compared to DCs exposed to vehicle (Fig. 4c, d). Furthermore, relative *iNOS* and *Il-6* mRNA expression was reduced when DCs were exposed to ROCK1/2-inhibitor compared to DCs exposed to vehicle under LPS stimulation (Fig. 4e, f). The observation that ROCK1/2-inhibition reduced *iNOS* production, which is an indicator of ROS production, is consistent with a role of ROS for tissue damage in aGVHD^5^. Conversely, *Il-10* mRNA expression, which exerts anti-inflammatory effects during aGVHD^37^, was increased in DCs exposed to ROCK1/2-inhibitor compared to DCs exposed to vehicle (Fig. 4g). DC migration is an essential process in the pathogenesis of aGVHD, as it was shown that DCs derived from the intestinal tract present antigen to donor T cells in mesenteric lymph nodes^38^. Therefore, we analyzed DC migration in a transwell assay and found that DC migration towards CXCL-12 was reduced when DCs were exposed to ROCK1/2-inhibitor compared to DCs exposed to vehicle (Fig. 4h). Modifications of the cytoskeleton are essential for cell migration which is reflected by phalloidin stained F-actin accumulation in the cell^39^. The abundance of phalloidin stained actin was reduced when DCs were exposed to ROCK1/2-inhibitor compared to DCs exposed to vehicle in vitro (Fig. 4i). In agreement, we found that the actin cytoskeleton of DCs from ROCK1/2-inhibitor treated mice had less actin structures at the cell surface and a thinner cortical actin cytoskeleton in vivo (Fig. 4j–m). To test if these in vitro effects of ROCK1/2 inhibition are relevant for the in vivo situation, we transferred DCs that had been pre-exposed to ROCK1/2-inhibitor. Utilizing the murine allo-HCT model (C57BL/6 into BALB/c), we observed an improved survival of mice that were injected with DCs exposed to ROCK1/2-inhibitor compared to mice receiving DCs exposed to vehicle (Fig. 4n). In addition, we found that transfer of DCs exposed to ROCK1/2-inhibitor was connected to lower aGVHD histopathology score of mice undergoing allo-HCT (C57BL/6 into BALB/c) compared to mice receiving DCs exposed to vehicle (Fig. 4o–q).

These findings indicate that ROCK1/2-inhibition has profound inhibitory effects on DCs including reduced stimulatory activity towards T cell proliferation, *Il-6* and *iNOS* production as well as migratory activity of DCs while anti-inflammatory *Il-10* was upregulated, which translates into a protective DC phenotype in vivo.

### ROCK1/2-inhibition reduces stimulatory activity, pro-inflammatory cytokine expression, and migratory activity of macrophages

**Fig. 5:**
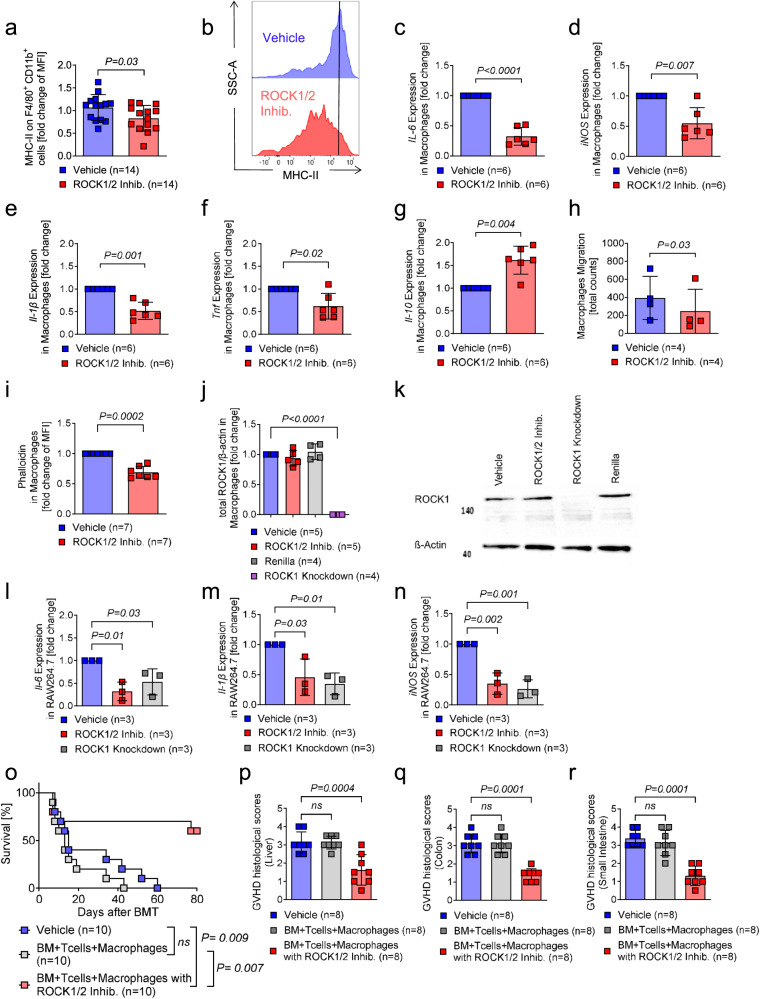
ROCK1/2-inhibition reduces MHC-II expression, *Il-6*, *iNOS*, and migratory activity of macrophages. **a** The scatter plot shows the fold change of MFI for MHC-II on F4/80^+^ CD11b^+^ cells on day 14 after allo-HCT. Mice underwent allo-HCT to induce aGVHD and were treated intraperitoneal with vehicle or ROCK1/2-inhibitor (8 mg/kg in 100 µl PBS) from day 4 to day 13. The experiment was performed three times, each point represents one mouse. The plot shows mean +/− SD and the *P*-value was calculated using a two-tailed unpaired student *t*-test. **b** Representative histogram showing MHC-II expression on F4/80^+^ CD11b^+^ cells. **c**–**g** BM-derived macrophages were stimulated with LPS for two hours and thereafter with ROCK1/2-inhibitor or vehicle for two hours. The scatter plots show the fold change of *Il-6* mRNA (**c**), *iNOS* mRNA (**d**), *Il-1β* mRNA (**e**), *Tnf* mRNA (**f**) and *Il-10* mRNA (**g**) isolated from BM-derived macrophages. Values of the experimental groups were normalized to control group. The experiment was performed three times, each point represents a mouse. The plot shows mean +/− SD and the *P*-value was calculated using a two-tailed paired student *t*-test. **h** The scatter plot shows the total count of macrophages migrated through a membrane towards MCP-1. BM-derived macrophages were pre-treated with LPS for two hours followed by ROCK1/2-inhibitor treatment or vehicle for two hours. Thereafter, they were incubated in a transwell-chamber for five hours with a MCP-1 gradient and migration was assessed by microscopy. The experiment was performed three times, each point represents one mouse. The plot shows mean +/− SD and the *P*-value was calculated using a two-tailed paired student *t*-test. **i** The scatter plot shows the fold change of MFI for phalloidin in BM-derived macrophages. Cells were treated with LPS for two hours and exposed to ROCK1/2-inhibitor or vehicle for two hours. The experiment was performed three times, each point represents a mouse. The plot shows mean +/− SD and the *P*-value was calculated using a two-tailed paired student *t*-test. **j** Quantification of ROCK1 in the LPS stimulated macrophage cell line RAW264.7 with ROCK1 knockdown or without (shRNA directed against Renilla luciferase) compared to BM-derived macrophages exposed to LPS and ROCK1/2-inhibitor or vehicle for two hours. The experiment was performed three times, each point represents a different passage of cells, values of the experimental groups were normalized to control group. The plot shows mean +/− SD and the *P*-value was calculated using a two-way ANOVA (Dunnett’s multiple comparison). **k** Representative western blot showing ROCK1 expression after ROCK1 knockdown or ROCK1/2-inhibitor treatment in RAW264.7 cells. The experimental design is the same as panel **j**. Uncropped western blots are shown in Source Data. **l**–**n** The scatter plots show the fold change of *Il-6* mRNA (**l**), *Il-1β* mRNA (**m**) and *iNOS* mRNA (**n**) isolated from RAW264.7 cells with a ROCK1 knockdown or without (shRNA directed against Renilla luciferase) compared to BM-derived macrophages exposed to LPS and ROCK1/2-inhibitor or vehicle for two hours. The experiment was performed three times, each point represents a different passage of cells, and values of the experimental group were normalized to control group. The plot shows mean +/− SD and the *P*-value was calculated using a two-way ANOVA (Dunnett’s multiple comparison). **o** Survival of mice undergoing allo-HCT to induce aGVHD. On the same day, BM-derived macrophages were incubated with ROCK1/2-inhibitor or PBS for four hours, thereafter 7 × 10^5^ donor macrophages were injected together with the graft. Survival of mice was plotted by using the Kaplan–Meier method and compared by using a log-rank (Mantel Cox) test. Depicted are 10 mice in each group from two individual experiments. **p**–**r** Histological aGVHD scoring of liver (**p**), colon (**q**) and small intestine (**r**) of mice on day 14 after allo-HCT as described in (**o**). The plots show mean +/− SD and the *P*-value was calculated using a two-way ANOVA (Dunnett’s multiple comparison).

Besides DCs, in previous reports^40^ donor monocyte-derived macrophages were also shown to promote human aGVHD. We observed that MHC-II expression on macrophages was reduced in mice that underwent allo-HCT when treated with ROCK1/2-inhibitor compared to mice treated with vehicle (Fig. 5a, b). In addition, *Il-6*, *iNOS*, *Il-1β*, and *Tnf* mRNA expression were reduced (Fig. 5c–f) whereas *Il-10* mRNA expression was upregulated (Fig. 5g) when macrophages had been exposed in vitro to ROCK1/2-inhibitor compared to macrophages exposed to vehicle. Immune cell migration is an essential step in the pathogenesis of aGVHD^11^. Therefore, we analyzed migration of macrophages in vitro when ROCK1/2 was inhibited. We observed that ROCK1/2-inhibition reduced macrophage migration (Fig. 5h), which was connected to reduced phalloidin-stained F-actin fibers (Fig. 5i). These findings are in line with previous studies showing that a reduction of F-actin fibers correlates with lower cell motility^39^. To test the role of ROCK1 in macrophages in a genetic approach, we induced a knockdown of ROCK1 using a doxycycline dependent system (Fig. 5j, k) in a macrophage cell line (RAW264.7). Upon stimulation with LPS, ROCK1 knockdown led to reduced transcription of *Il-6*, *Il-1β*, and *iNOS* in macrophages (Fig. 5l–n). Furthermore, we found that the transfer of ROCK1/2 pre-exposed macrophages to mice undergoing allo-HCT was connected to an improved survival and lower aGVHD histopathology scores compared to mice receiving macrophages exposed to vehicle (Fig. 5o–r). These findings indicate that ROCK1/2-inhibition has inhibitory effects on macrophages including reduced pro-inflammatory cytokine production as well as migratory activity, which translates into a protective macrophage phenotype in vivo.

### ROCK1/2-inhibition alters the gene expression profile in vivo and reduces NF-κB and cofilin phosphorylation in DCs and in macrophages in vitro

**Fig. 6:**
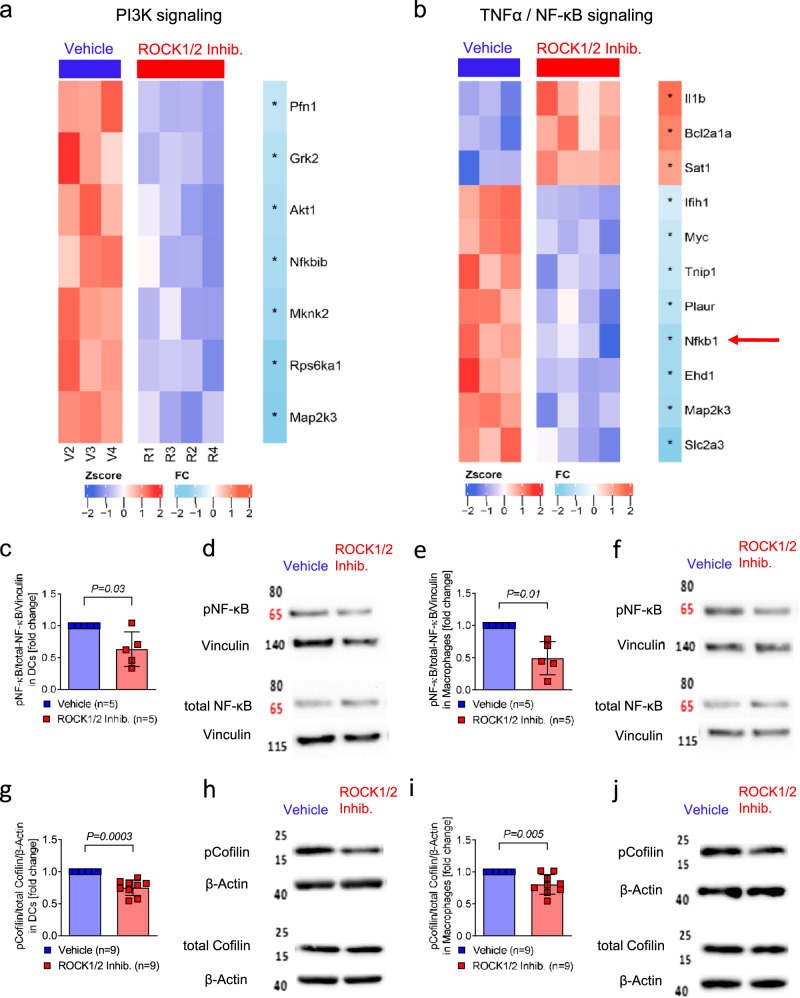
ROCK1/2-inhibition alters the gene expression profile in vivo and reduces NF-κB and cofilin phosporylation in DCs and in macrophages in vitro. **a** The heatmap shows the expression of downregulated genes related to PI3K signaling in CD45^+^CD11b^+^ cells that were FACS-isolated from mice on day 14 after allo-HCT after 10 days of ROCK1/2-inhibitor or vehicle treatment. Color scale represents the row-wise z-score intensity. * Indicates significant changes in gene expression. **b** The heatmap shows the expression of genes involved in *Tnf* signaling of CD45^+^CD11b^+^ cells isolated as indicated in (**a**). Color scale represents the row-wise z-score intensity. * Indicates significant changes in gene expression. Red arrow indicates NF-κB1 gene. **c**–**f** The western blots show total and phosphorylated NF-κB of BM-derived DCs or macrophages incubated with LPS for two hours and thereafter with ROCK1/2-inhibitor for two hours. Samples derived from the same experiment and gels/blots were processed in parallel. Loading controls ran on the same blot. When applicable, data is normalized to control group, the experiments were performed three times, each point represents a mouse, values of the experimental group were normalized to control group. The plots show mean +/− SD and the *P*-value was calculated using a two-tailed paired student *t*-test. Uncropped western blots are shown in Source Data. **c** The bar graph shows pNF-κB/total NF-κB in BM-derived DCs. **d** Representative western blot image of pNF-κB and total NF-κB in BM derived DCs. **e** The bar graph shows pNF-κB/total NF-κB in BM-derived macrophages. **f** Representative western blot image of pNF-κB and total NF-κB in BM-derived macrophages. **g**–**j** The western blots show total and phosphorylated Cofilin of BM-derived DCs or macrophages incubated with LPS for two hours and thereafter with ROCK1/2-inhibitor for two hours. Samples derived from the same experiment and gels/blots were processed in parallel. Loading controls ran on the same blot. When applicable, data are normalized to control group, the experiments were performed three times, each point represents a mouse, values of the experimental group were normalized to control group. The plots show mean +/− SD and the *P*-value was calculated using a two-tailed paired student *t*-test. Uncropped western blots are shown in Source Data. **g** The bar graph shows pCofilin/total Cofilin in BM-derived DCs. **h** Representative western blot image of pCofilin and total Cofilin in BM-derived DCs. **i** The bar graph shows pCofilin/total Cofilin in BM-derived macrophages. **j** Representative western blot image of pCofilin and total Cofilin in BM-derived macrophages.

To better understand the effects of ROCK1/2-inhibition on myeloid cells at the signaling level, we isolated CD11b^+^ cells on day 14 after allo-HCT from mice that were treated with ROCK1/2-inhibitor or vehicle and then studied their gene expression. We observed downregulation of *PI3K* and *TNF/NF-κB* signaling-related gene expression (Fig. 6a, b). Both *PI3K* and *TNF /NF-κB* signaling had been shown to be involved in aGVHD^11^. Therefore, we next analyzed the impact of ROCK1/2-inhibition on *NF-κB* signaling in detail. The phosphorylation of NF-κB relative to total NF-κB declined in DCs (Fig. 6c, d) and macrophages (Fig. 6e, f) upon ROCK1/2-inhibition in vitro. These findings indicate that ROCK1/2-inhibition interferes with major aGVHD-related pathways. An indicator for ROCK1 activity is cofilin phosphorylation, which we found to be reduced upon ROCK1/2-inhibition indicating on target activity in DCs and macrophages (Fig. 6g–j).

### ROCK1/2-inhibition allows for cytotoxicity and GVL effects in mice and has additive effects with JAK1/2-inhibition

**Fig. 7:**
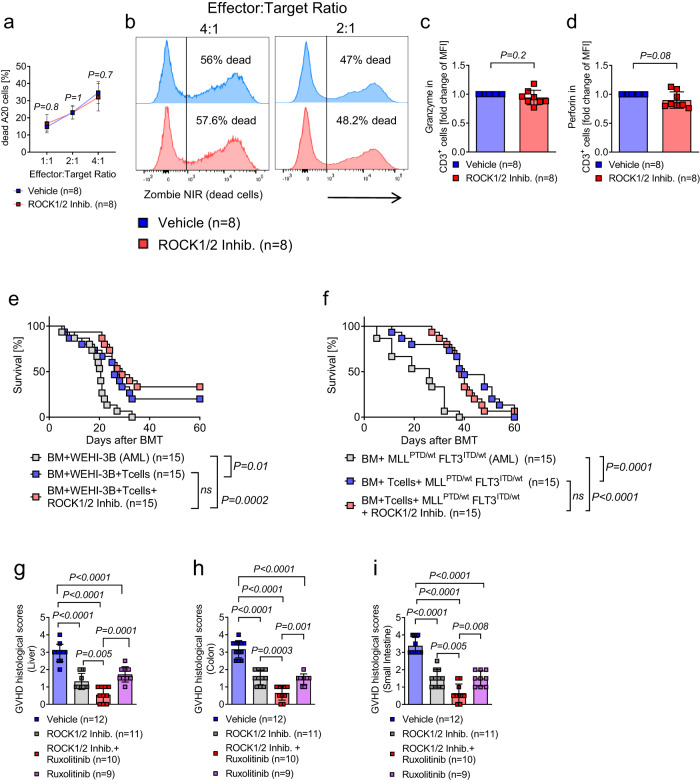
ROCK1/2-inhibition allows for cytotoxicity and GVL effects in mice and has additive effects with JAK1/2-inhibition. **a** The diagram shows the percentage of dead A20 cells that had been exposed to allogeneic T cells at different ratios (effector to target 1:1, 2:1, 4:1). Prior to the coculture, T cells were preincubated with ROCK1/2-inhibitor for two hours. T cells were activated with CD3/CD28 Dynabeads for 48 h. Cytotoxicty was measured by FACS after 20 h of coculture. Represented data are pooled from three independent experiments performed in triplicate. The plots show mean +/− SD and the *P*-value was calculated using an ordinary two-way ANOVA test (Sidaks multiple comparison). **b** Representative histograms showing the percentage of dead A20 cells of all A20 cells after exposure to allogeneic T cells as described in panel (**a**). **c** The scatter plot shows the fold change of granzyme B in CD3^+^ cells. T cells were activated with CD3/CD28 Dynabeads for 48 h and then exposed to ROCK1/2-inhibitor for two hours. The experiment was performed three times, each point represents a mouse, values of the experimental group were normalized to control group. The plots show mean +/− SD and the *P*-value was calculated using a two-tailed paired student *t*-test. **d** The scatter plot shows the fold change of perforin in CD3^+^ cells. The experimental design was the same as panel (**c**). **e** Survival of mice after allo-HCT that received WEHI-3B leukemia cells and BM cells alone or BM/T cells. ROCK1/2-inhibitor was injected from day 4 to day 13. Survival was plotted using the Kaplan–Meier method and compared by using a log-rank (Mantel Cox) test. The experiment was performed three times, each point represents a mouse (15 mice in each group). **f** Survival of mice after allo-HCT that received MLL^PTD/wt^ FLT3 ^ITD/wt^ AML cells and BM cells alone or BM/T cells. ROCK1/2-inhibitor was injected from day 4 to day 13. Survival was plotted using the Kaplan–Meier method and compared by using a log-rank (Mantel Cox) test. The experiment was performed three times, each point represents a mouse (15 mice in each group). **g**–**i** Histological aGVHD scoring of liver (**g**), colon (**h**) and small intestine (**i**) of mice on day 14 after allo-HCT that received either ROCK1/2-inhibitor, ruxolitinib, a combination of ROCK1/2-inhibitor and ruxolitinib or vehicle treatment as described before. The plots show mean +/_ SD and the *P*-value was calculated using a two-way ANOVA (Dunnett’s multiple comparison).

Since ROCK1/2-inhibition had inhibitory effects on multiple immune cell types and pro-inflammatory signaling, we next analyzed if GVL effects were affected by ROCK1/2-inhibition. To clarify this, T cells were activated with CD3/CD28 beads, exposed to ROCK1/2-inhibitor for two hours thereafter and cultured together with allogeneic A20 lymphoma cells for 20 h. Cytotoxicity increased with increasing effector cell ratios independent of treatment with ROCK1/2-inhibitor (Fig. 7a, b). The cytotoxic markers granzyme B and perforin were equally expressed in T cells exposed to vehicle or ROCK1/2-inhibitor (Fig. 7c, d). To test the GVL effect in vivo we used well-established leukemia models^41,42^. We observed an improved survival of WEHI-3B AML-bearing mice (BALB/c recipients) that were injected with allogenic BM and T cells (C57BL/6) compared to those that were injected with allogeneic BM only (Fig. 7e). We did not find any difference in the survival when the mice were additionally treated with ROCK1/2-inhibitor or vehicle (Fig. 7e). To confirm these findings, we then used a second GVL mouse model relying on the injection of MLL^PTD/wt^ FLT3 ^ITD/wt^ AML cells. Similarly, ROCK1/2-inhibitor treatment did not alter the ability of T cells to exert GVL effects (Fig. 7f).

Since ruxolitinib is approved for patients with SR-aGVHD, we next aimed to clarify if ROCK1/2-inhibition and JAK1/2-inhibition act in synergy. Utilizing our murine allo-HCT model, we observed an improvement in histological GVHD scores with an additive effect of ruxolitinib and Y-27632 compared to single drug therapy (Fig. 7g–i).

**Fig. 8:**
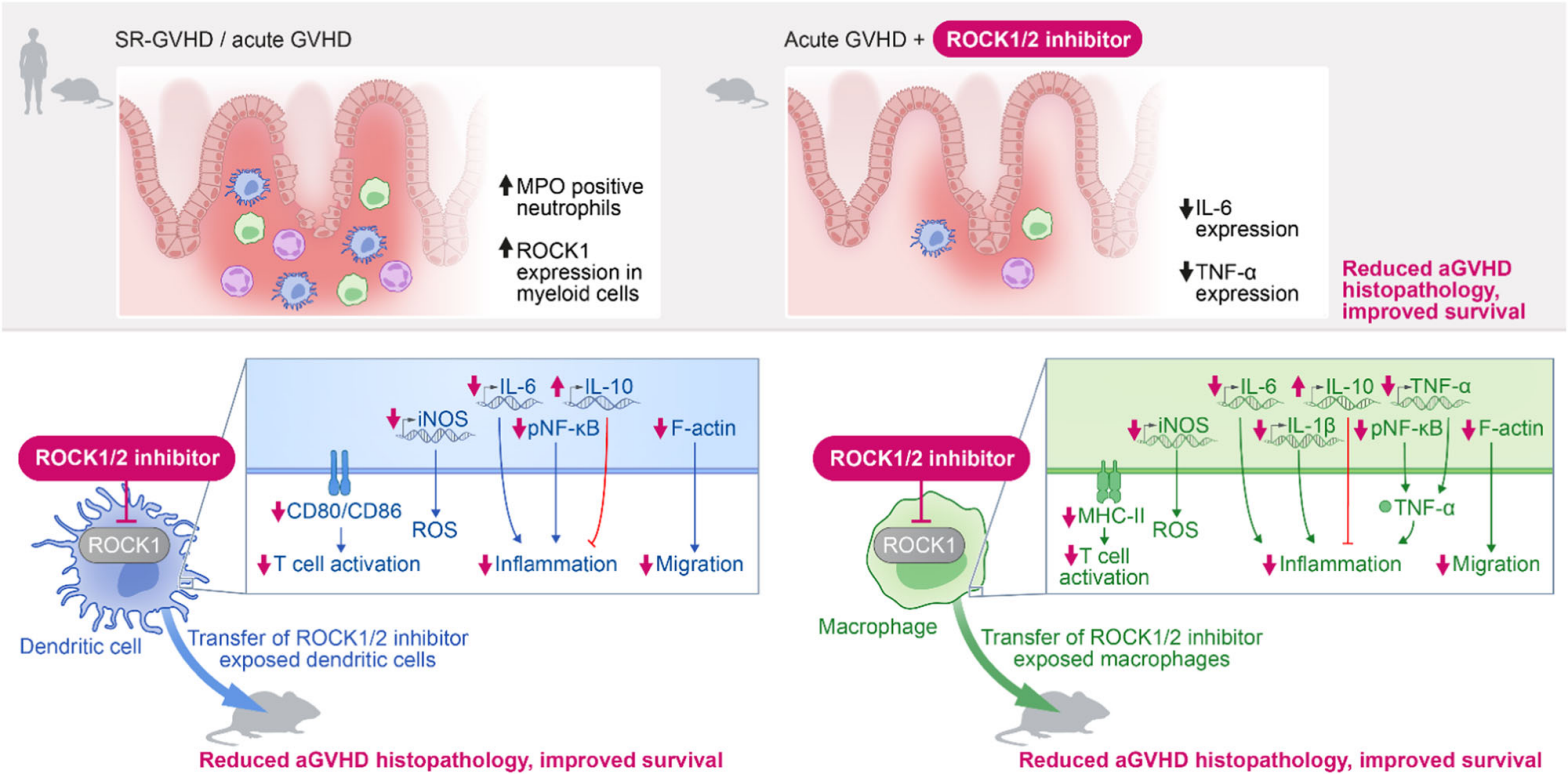
Proposed mechanism of action of ROCK1 inhibition. Acute GVHD pathophysiology (left panel) involves expansion of MPO positive neutrophils and upregulation of ROCK1 expression in myeloid cells. ROCK1/2-inhibition in mice leads to reduced IL-6 and TNF levels, thereby reducing aGVHD histopathology and improving survival. ROCK1/2-inhibition counteracts multiple pathogenic events in dendritic cells (blue panel) and macrophages (green panel) including TNF production by DCs and macrophages, antigen presentation and migration. Moreover, ROCK1/2-inhibition in macrophages or dendritic cells prior to transfer reduces aGVHD severity. On the signaling level ROCK1/2-inhibition interferes with the NF-κB pathway activity.

Together, these findings show that ROCK1/2-inhibition reduces aGVHD without impairing GVL effects. Moreover, our findings indicate additive effects against aGVHD when combined with JAK1/2-inhibition (Fig. 8).

## Discussion

Acute GVHD remains a major life-threatening complication after allo-HCT, indicating that alternative treatment strategies are needed. Kinases are promising therapeutic targets to treat acute^11^ and chronic^43^ GVHD because inhibiting a single kinase can interfere with the downstream effects of multiple cytokine receptors. Different kinase inhibitors have been developed from the mouse model into FDA approved GVHD therapies, such as ibrutinib, belumosudil and ruxolitinib for SR-cGVHD^28,44,45^ and ruxolitinib for SR-aGVHD^34,35,46,47^. Since SR-aGVHD represents a major unmet clinical need, we applied a kinase enrichment approach to discover novel drug targets in this disease. This unbiased proteomics-based analysis led to the discovery of high levels of activated ROCK1 in leukocytes of SR-aGVHD patients, which was confirmed by flow-cytometry. The role of the ROCK1 kinase has so far not been investigated in aGVHD. Conversely, ROCK2-inhibition has been shown to be beneficial in a cGVHD mouse model^27^ and to be effective and safe in the clinical trial setting^28,48^ and was approved for cGVHD^49^. Prophylactic treatment with Fasudil, another ROCK1/2 inhibitor, reduces aGVHD^50^. In line with this work, we also demonstrate in this study that ROCK1/2-inhibition reduced aGVHD-related death and intestinal aGVHD histopathology. Moreover, our findings extend these results since we show in detail that ROCK1/2 inhibition exerts various effects on DCs and macrophages in vitro and in vivo.

Different groups have shown that prior to the expansion of alloreactive cytotoxic T cells, irradiation or chemotherapy-based conditioning regimen led to the recruitment and activation of neutrophils^5,51,52^ and monocytes^7^ in mouse models of aGVHD. In these studies both donor monocyte-derived macrophages^40^ and donor DCs^53^ can induce GVHD. Donor DCs migrate to lymph nodes, drive T cells expansion and produce pro-inflammatory cytokines like Il-6 and Il-12^38^. Consistently with these reports, we found that ROCK1/2-inhibition of donor-derived DCs and macrophages improved survival of mice after allo-HCT. Moreover, ROCK1/2-inhibition reduced the production of *iNOS*, which is connected to reduced production of ROS, previously shown to damage the intestinal barrier when released by neutrophils residing in the intestinal wall after allo-HCT^5,52^.

The ability of DCs to stimulate T cell proliferation and to produce the pro-inflammatory cytokine *Il-6* was reduced upon ROCK1/2 inhibition, whereas the production of *Il-10* was increased. Regarding macrophages, we found that ROCK1/2-inhibition reduced pro-inflammatory cytokine expression and MHC-II expression. Moreover, ROCK1 knockdown in a macrophage cell line also caused reduced inflammatory cytokine production, while upregulating the production of *Il-10*. These data are consistent with work demonstrating an improvement of aGVHD when M2 macrophages instead of M1 macrophages were transferred into GVHD mice^54^.

ROCK1 has been found to promote migration of different cell types, among them non-small-cell lung cancer cells^55^ and retinal glial cells^56^. Migration of neutrophils and pro-inflammatory monocytes to the site of tissue damage is an important step to promote inflammation in aGVHD. In agreement, we observed that migration of myeloid cells was reduced when ROCK1/2 was inhibited. Furthermore, we found that actin structures were modified upon ROCK1/2 inhibition both in vitro and in vivo. These findings support the concept that multiple layers of the immune response are affected by ROCK1/2 inhibition.

On the signaling level we found that ROCK1/2-inhibition reduced the expression of genes related to *IL-6* and *JAK-STAT3* signaling and the phosphorylation of NF-κB in macrophages and DCs. JAK-STAT3 and NF-κB signaling were both shown to promote the activity of the CIITA/MHC-II axis^57^.

Manipulation of the immune response could lead to loss of GVL effects^41^. However, we observed that ROCK1/2-inhibition did not reduce GVL effects and did not affect alloantigen driven cytotoxicity of T cells in vitro.

JAK1/2 inhibition has become the gold standard for treatment of SR-cGVHD^45^. Intriguingly, ROCK1/2-inhibition acted synergistically with JAK1/2-inhibition in vivo, which is important for future clinical study design.

In summary, we identified ROCK1 as a highly upregulated kinase in SR-aGVHD patients. Using a mouse model of aGVHD, we provided evidence that ROCK1/2-inhibition diminishes allo-immune activation at different cellular and molecular levels without a loss of GVL activity. Based on our study, ROCK1/2-inhibition, which is already used in clinical trials for pulmonary arterial hypertension^58^ and coronary stenosis^59^, is a promising approach to treat acute SR-GVHD, possibly in combination with ruxolitinib.

## Methods

### Human tissue analysis

The study included formalin-fixed and paraffin-embedded (FFPE) liver, colon, and small bowel tissue specimens (Supplementary Table 1) for histology. PBMCs (Supplementary Tables 2, 3) were used for analysis via mass spectrometry from patients that underwent allo-HCT for the treatment of different hematological malignancies. We obtained written informed consent from all patients prior to analysis. The studies were conducted in accordance with the ethical guidelines of the Declaration of Helsinki and were approved by an institutional review board, and by the local ethics committee (protocol no.: 547/14; Ethic committee, Albert-Ludwigs-University, Freiburg, Germany), the non-interventional study for the patient sample collection was registered at ClinicalTrials.gov (Identifier: NCT04342442).

Patients who underwent allo-HCT at the University hospital Freiburg were included in the study. Patients with aGVHD were defined as non-responsive to corticosteroids (SR-aGVHD) if they had received prednisolone at a dose of 2 mg/kg bodyweight for at least 7 days without response or when aGVHD progression was documented after five days of treatment and the clinical situation required additional immunosuppressive therapy. As indicated, the control groups consisted (a) of patients who had aGVHD responsive to corticosteroids and achieved at least a partial remission to steroids, (b) patients post allo-HCT who never developed aGVHD or (c) healthy controls.

### PBMC sample preparation

Blood was collected at multiple time points of the same treatment regimen. Samples acquired during different aGVHD treatment regimens from the same patient were treated as independent samples. Blood samples were diluted 1:3 with PBS, layered onto Pancoll and centrifuged (30 min, 440 × *g*) without break. For FACS analysis one volume of EDTA-blood was mixed well with 19 volumes of pre-warmed (37 °C) BD Phosflow™ Lyse/Fix Buffer (5X concentrate diluted to 1X with deionized water) and was then incubated at 37 °C for 10 min. Cells were centrifuged (8 min, 500 × *g*) and the pellet was washed twice with HBSS without Ca^2+/^Mg^2+^. Cells were re-suspended in freezing solution (90% FACS buffer consisting of PBS, EDTA 2 mM, BSA 0.5% + 10% DMSO), stored overnight at −80 °C and then transferred into liquid nitrogen.

### Mice

C57BL/6 (H-2K^b^) and BALB/c (H-2K^d^) mice were purchased from Janvier Labs (France) or from the local stock of the animal facility at University of Freiburg. Mice were used between 6 and 14 weeks of age, and only female or male donor/recipient pairs were used. Animal protocols were approved by the animal ethics committee Regierungspräsidium Freiburg, Freiburg, Germany (No: G-22/103, G17/63, G18/36, G20/078, G22/007).

### Isolation of BM-derived macrophages and DCs

Femora and tibiae were taken from C57BL/6(H-2K^b^) mice and 3.5–6 × 10^6^/10 ml naive bone marrow cells were isolated in placed into culture. mM-CSF or mGM-CSF were added to the culture and cells were incubated at 37 °C and 5% CO_2_ for one week.

### Bone marrow (BM) transplantation model

BM transplantation experiments were performed as described^5^. The major mismatch strain combinations used were C57BL/6 into BALB/c or BALB/c into C57BL/6 as indicated in the respective experiments. Briefly, recipients were injected by intravenous injection (i.v.) via tail vein with 5×10^6^ BM cells after lethal irradiation with 1000 cGy (BALB/c recipient) or 1200 cGy (C57BL/6 recipient), using a ^137^Cs source split into two equal doses and four hours apart. To induce aGVHD, CD4^+^ and CD8^+^ T cells were isolated from donor spleens and enriched with the MACS cell separation system and the Pan T Cell Isolation Kit II (Miltenyi Biotec, Germany) according to the manufacturer’s instructions. CD4^+^/CD8^+^ T cells were given at a dosage of 0.3 – 0.5×10^6^ on day 0 as indicated in the respective experiments.

### Graft-versus-Leukemia models

We used splenocytes from mice carrying the mutations MLL-PTD and FLT3-ITD as a primary acute myeloid leukemia (AML) model. Lethally irradiated (1200 cGy) C57BL/6J recipient mice were transplanted with 5000 MLL^PTD/wt^ FLT3^ITD/wt^ AML cells and 5 × 10^6^ BALB/c BM cells were injected i.v. into the tail vain. On day 2 after allo-HCT, 0.3 × 10^6^ MACS-purified donor CD4^+^ and CD8^+^ T cells were injected i.v. to respective groups. As a second model, the murine AML cell line WEHI-3B was used. Lethally irradiated (1000 cGy) BALB/c recipient mice were transplanted i.v. with 5 × 10^6^ BM cells and 5000 WEHI-3B AML cells. On day 2 after allo-HCT, 0.3 × 10^6^ MACS-purified donor CD4^+^ and CD8^+^ T cells were injected i.v. to respective groups.

### ROCK1/2-inhibitor treatment

The ROCK1/2-inhibitor Y-27632 (Tocris) was diluted in PBS and given once per day per intraperitoneal (i.p.) injection at a dose of 8 mg/kg bodyweight or an equivalent of PBS to mice. The dosage was based on reports^60,61^. Y-27632 is a selective ROCK1 (p160ROCK) inhibitor with a Ki of 140 nM. At higher concentrations ROCK2 is also inhibited. The duration of the treatment is indicated for each experiment. For GVL and aGVHD experiments treatment was from day 4 to day 13.

### Ruxolitinib + ROCK1/2-inhibitor treatment

Ruxolitinib (INCB18424, MedChemExpress) was dissolved in PEG300/dextrose 5% in a ratio 1:3 (PEG/dex) and given by oral gavage at a dosage of 30 mg/kg two times daily starting from day -1 to day 13 after allo-HCT. The ROCK1/2-inhibitor Y-27632 was diluted and given as previously described from day 4 to day 13. The control group received PEG/dex and PBS alone.

### In vitro treatment of macrophages and dendritic cells

To stimulate the cells in vitro, 250 ng/ml LPS was added to the cells for two hours. After incubation, 30 µg/ml ROCK1/2-Inhibitor Y-27632 was added for two hours. Thereafter, cells were washed and measurements (e.g. qPCR) were performed.

### aGVHD histopathological scoring

To determine the histopathological severity of aGVHD, we obtained sections from the liver, small intestine and colon of the recipient mice. Those samples were stained with hematoxylin and eosin. Based on a published histopathologic scoring system^33^, the samples were scored by an experienced pathologist who was blinded to the experimental groups.

### Microarray analysis

Splenic CD11b^+^ cells were isolated from recipient mice on day 14 after allo-HCT using FACS sorting. Total RNA was isolated from cells using a Qiagen miRNeasy Mini Kit. RNA quality was assessed using an Agilent 2100 Bioanalyzer (Agilent Technologies). Microarray analysis was performed using Affymetrix Clariom S Mouse arrays. Arrays were normalized using Robust Multi-Array Average expression measure^62^_,_ and probe expression values summarized to the gene level using the R/Bioconductor package pd.clariom.s.mouse. The complete data are available in the GEO repository under the access ID.

### Differential analysis

A linear model based approach (limma R package)^63^ was used to identify the differentially regulated RNA between untreated and treated cells. Regulated RNA with Benjamini Hochberg adjusted *P-*value < 0.05 was set as significant.

### Statistical analysis

For statistical analysis, an unpaired or paired student *t*-test (two-sided) or when required a two-way ANOVA (with Dunnett’s multiple-comparison test) was applied. If the data did not meet the criteria for normality, the Mann–Whitney *U* test was applied unless stated otherwise in the Figure legend. Data are presented as mean and +/− SD (error bars). Differences were considered significant when the *P*-value was <0.05. Survival data were plotted using the Kaplan–Meier method and compared by using a Log-rank (Mantel–Cox) test.

### Flow cytometry

Samples were washed in PBS and dead cells were stained using either Aqua LiveDead (Thermo Fisher Scientific) or Zombie NIR fixable dye (Biolegend). Fc receptors were blocked using anti-CD16/CD32 (1:100 dilution), before staining with appropriate antibody concentrations for 20 min at 4 °C. In some cases, cells were fixed in 0.5% PFA. For analysis of intracellular antigens cells were fixed in Fix/Perm (eBioscience) for 30 min at 4 °C and thereafter incubated with intracellular antibodies for 60 min at 4 °C. Data was acquired within two hours after staining on a BD LSR Fortessa (BD Biosciences) and analysis performed using FlowJo software (FlowJo, LLC). For Umap and FlowSOM plots, data was exported from FlowJo and uploaded into Rstudio (Posit). High dimensional analysis was performed as described^64^. Antibodies for flow cytometry and microscopy are listed in Supplementary Table 4.

Absolute counts of neutrophils, monocytes, and lymphocytes were calculated based on the frequencies in the acquired samples and the absolute counts of leukocytes in fresh blood before fixation.

### Kinase enrichment using Kinet-1 beads

Protein was isolated from human PBMC derived from patients who were diagnosed with acute GVHD grade 3-4 and had failed to respond to 2 mg per kg bodyweight prednisolone (Decortin H) after at least five days of treatment, or when indicated PBMC were from patients not affected by GVHD (*n* = 6 per group plus one technical replicate of one individual per group). The protein was then exposed to Kinet-1 beads as previously described^41^. PBMC samples were collected from each patient at different time points of the same treatment period and frozen at −80 °C. Kinase enrichment was performed as previously described^29^ with adaptations. Briefly, the cells were lysed 30 min on ice in lysis buffer (50 mM HEPES-NaOH pH7.5, 150 mM NaCl, 0.5% Triton-X 100, 1 mM EDTA, 1 mM EGTA) supplemented with 10 mM NaF, 2.5 mM Na_3_VO_4_, PhosSTOP^TM^ phosphatase inhibitor cocktail and cOmplete^TM^ protease inhibitor cocktail (Roche, Mannheim, Germany) and centrifuged for 30 min at 16,000 × *g*. Lysates of each patient sample were pooled considering the treatment periods. The concentration was measured using Bradford Assay (Kit Thermo Fisher Scientific) and adjusted to 2 mg/mL, and the NaCl concentration was adjusted to 1 M. One mL of lysate was incubated with 35 µL bead-bound pan-kinase inhibitor (Kinet-1, SYNkinase) at 4 °C for 3 h on a rotating wheel. With each of the different washing buffers, three washing steps were performed (Buffer 1: lysis buffer stock + 1 M NaCl, 10 mM NaF, 0.1 mM Na_3_VO_4_; Buffer 2: lysis buffer stock + 10 mM NaF, 0.1 mM Na_3_VO_4_; Buffer 3: 50 mM HEPES-NaOH (pH = 7.5), 10 mM NaF, 0.1 mM Na_3_VO_4_) and the beads were frozen at −80 °C.

### Kinase enrichment approach and proteomics analysis

Proteins were eluted in SDS loading buffer containing 1 mM DTT for 10 min at 75 °C. After alkylation using 5.5 mM iodoacetamide for 10 min at room temperature the samples were centrifuged and the supernatants were loaded on 4–12% gradient gels (NuPAGE, Thermo Fisher) for protein separation. After staining, each gel lane was cut into 7 slices, the proteins were in-gel digested with trypsin (Promega) and the resulting peptide mixtures were processed on STAGE tips and analyzed by LC-MS/MS essentially as described^65^. The LC-MS measurements were performed on a QExactive Plus mass spectrometer coupled to an EasyLC 1000 nanoflow-HPLC. Peptides were separated on fused silica HPLC-column tip (I.D. 75 µm, New Objective, self-packed with ReproSil-Pur 120 C18-AQ, 1.9 µm (Dr. Maisch) to a length of 20 cm) using a gradient of A (0.1% formic acid in water) and B (0.1% formic acid in 80% acetonitrile in water): loading of sample with 0% B with a flow rate of 600 nl/min; separation ramp from 5–30% B within 85 min with a flow rate of 250 nl/min). For nanoESI the spray voltage was set to 2.3 kV and ion-transfer tube temperature to 250 °C, no sheath and auxiliary gas was used. The mass spectrometer was operated in the data-dependent mode; after each MS scan (mass range *m/z* = 370–1750; resolution: 70,000) a maximum of ten MS/MS scans were performed using a normalized collision energy of 25%, a target value of 1000 and a resolution of 17,500. The MS raw files were analyzed using MaxQuant Software version 1.4.1.2^66^ for peak detection, quantification and peptide identification using a full length UniProt human database (March, 2016) and common contaminants such as keratins and enzymes used for in-gel digestion as reference. Carbamidomethylcysteine was set as fixed modification and protein amino-terminal acetylation, deamidation of asparagine and glutamine, pyro-glutamate and glutamine, and oxidation of methionine were set as variable modifications. The MS/MS tolerance was set to 20 ppm and three missed cleavages were allowed using trypsin/P as enzyme specificity. Peptide and protein FDR based on a forwards-reverse database were set to 0.01, minimum peptide length was set to 7, and minimum number of peptides for identification of proteins was set to one, which must be unique. The “match-between-run” option was used with a time window of 2 min. Proteins were quantified label-free using the iBAQ algorithm^67^ with a minimum ratio count of two.

For data analysis the Perseus computational platform was used^68^. Briefly, quantified proteins were filtered for kinases and only kinases identified in all 14 samples (6 samples per group plus one technical replicate) were further considered. Log2 transformed iBAQ values were used to identify significantly enriched kinases comparing GVHD to non-GVHD samples using a two-sided *t*-test, FDR 0.05, S0 = 0.1.

### F-actin staining for microscopy

Cells were pretreated with ROCK1/2-inhibitor as previously described and were then transferred to coverslips and washed in PBS before incubation with 4% formaldehyde solution (without methanol) for 25 min at RT. After fixation cells were washed in PBS and incubated with Alexa Fluor 488^TM^ Phalloidin for 60 min at RT. Subsequently, cells were washed again to remove excess dye. Super resolution images of the actin cytoskeleton (F-actin) were acquired with an ELYRA 7 structured-illumination microscope (3D Lattice-SIM) equipped with a ×63/1.4 objective (Carl Zeiss Microscopy Deutschland GmbH). Cells were imaged as z-stacks with a 0.11 µm interval. For SIM reconstruction by Zen Black, the automated Wiener filter strength given by the ‘standard’ end criterion of the manufacturer was used. Cells were visualized in 3D with Imaris software (Oxford Instruments) and for F-actin quantification the surface-function was applied.

### Western blot analysis

For Western blot analysis, cells were pretreated with ROCK1/2-inhibitor as described above and were then lysed with radioimmuno precipitation assay buffer (Santa Cruz Biotechnology) supplemented with Phosphatase Inhibitor Cocktail 2 (Sigma-Aldrich). Using Pierce™ Bicinchoninic Acid (BCA) Protein Assay Kit (Thermo Fischer Scientific), total protein concentrations were determined. Proteins were separated by electrophoresis on a 4–12% Bis-Tris NuPAGE™ gel (Thermo Fisher Scientific), then transferred to Amersham™ Protan™ 0,45 µm nitrocellulose membranes (Ge Healthcare Life science). Antibodies are listed in Supplementary Table 5. Antibodies were diluted in 5% nonfat dry milk solution or in 5% BSA in TBS-T. The blot signal was detected using WesternBright Quantum kit (Advansta Inc., San Jose) and imaged using the INTAS Science Imaging ECL Chemostar (INTAS Science Imaging Instruments GmbH). Quantification of Western blots was done using LabImage 1D INTAS software. Uncropped Western blots are provided in Source Data.

### qRT-PCR

Quantitative polymerase chain reaction was used to measure the expression of *iNOS*, *Il-6*, *Tnfα*, *Il-10*, and *Il-1β*. Therefore, RNA from dendritic cells and macrophages, pretreated as previously described were isolated by using RNeasy Mini KIT (Qiagen) according to the manufacturer´s protocol. Isolated RNA was transcribed into single-stranded complementary DNA (cDNA) by using High-Capacity cDNA RT Kit (Thermo Fisher scientific) according to the manufacturer´s protocol. qRT-PCR was performed using Power SYBR Green PCR Master Mix (Thermo Fisher Scientific) and experiments were run on a Roche LightCycler® 480 system (Roche Germany Holding GmbH). Each sample was measured in triplicate and gene expression was normalized to the expression of reference genes glyceraldehyde-3-phosphate dehydrogenase (GAPDH) and hypoxanthine phosphoribosyl transferase (HPRT) by means of ΔCt method and expressed relative to the LPS stimulated group (ΔΔCT). Primer sequences are listed in Supplementary Table 6.

### Transwell migration assay

Respective dendritic cells or macrophages were treated for two hours with LPS, followed by two hours of  30 µg/mL ROCK1/2-inhibitor Y-27632 or PBS. Afterwards, 1 × 10^5^ cells were seeded in the inserts of Corning Transwell Plates (Corning, 3 µm or 5 µm pore size) and added to the plate containing RPMI-1640 with 2% FCS and 60 ng/mL CXCL12 or MCP-1. After five hours of incubation at 37 °C under a 5% CO_2_ atmosphere, the number of migrated semi-adherent cells like dendritic cells was quantified by flow cytometry. Due to their adherent nature, macrophages were fixed on the membrane with 70% ethanol for 10 min and thereafter stained with 0.2% crystal violet solution for 10 min. Migration was measured visually by counting cells in five different fields of view under Axiovert 25 inverse Microscope (Carl Zeiss Microscopy Germany) at 100× total magnification.

### Coculture of dendritic cells and T cells

Bone marrow-derived DC were generated as previously described^57^. On day 6, dendritic cells were stimulated with 5 ng/mL LPS for 24 h. On day 7, cells were incubated with 30 µg/mL ROCK1/2-inhibitor Y-27632 for two hours. Splenic T cells of C57BL/6 (H-2k^b^) mice were MACS purified using the Pan T Cell Isolation Kit II (Miltenyi Biotec) and stained with CellTrace™ Violet (Invitrogen). T cells were labeled with CellTrace^TM^ Violet (Invitrogen) at a concentration of 2 µM for 20 min at 37° in prewarmed PBS. The reaction was stopped with RPMI-1640 medium including 10% fetal calf serum (FCS). Afterward cells were washed with PBS, resuspended in proliferation medium (containing 10% FCS, 1% P/S, 25 µM β-mercaptoethanol, 0.6 µl/ml IL-2).

0.1 × 10^6^ dendritic cells and 0.1 × 10^6 ^T cells were seeded together and cocultured for 72 h at 37 °C and 5% CO_2_.

To determine cell populations, anti-CD11c, anti-CD8, anti-CD4, anti-CD80, anti-CD86 were stained and data were acquired by flow cytometer BD LSR Fortessa (BD Bioscience). Data analysis was performed using FlowJO software 11 (FlowJo,LLC, BD Science).

### Cytotoxicity assay

Splenic T-cells of C57BL/6 (H-2k^b^) mice were MACS purified using the Pan T Cell Isolation Kit II (Miltenyi Biotec) and activated with CD3/CD28 Dynabeads™ (Thermo Fisher Scientific) for three days in a 96 well plate (1 × 10^5^/well). On day 4 the beads were washed off according to the protocol and T cells were incubated with 30 µg/mL ROCK-inhibitor Y-27632 for two hours. After washing, T cells were incubated with CellTrace™ Violet (Invitrogen) labeled A20 lymphoma cells (ratio 1:1, 2:1, 4:1) for 20 h. Dead A20 cells (Zombie NIR^TM^ CD19^+^ CellTrace^TM+^ Violet) were acquired by flow cytometer BD LSR Fortessa (BD Bioscience). Data analysis was performed using FlowJO software 11 (FlowJo,LLC, BD Science).

### ROCK1 knockdown

Different shRNAs directed against the mRNA of *Rock1* were designed using the webtool SplashRNA^69^. The shRNA oligonucleotide sequences are listed in Supplementary Table 7. The oligonucleotides were custom synthesized (Sigma-Aldrich) and PCR amplified for introduction of a *BspQI* restriction site. For knockdown experiments with inducible shRNAs, the amplified oligonucleotides were cloned into the recipient pMSCV-**T**RE-ds**R**ed-miR**E**-PGK-**B**SD-P2**A**-**V**enus (TREBAV) vector by restriction digest with *SapI* (Thermo Fisher scientific). As a control for knockdown experiments, a shRNA directed against *Renilla* luciferase (Supplementary Table 7) was used. For knockdown experiments with constitutive shRNA expression, the miRE cassette containing the respective shRNA was excised from the TREBAV vector using the endonucleases *BglII* (Thermo Fisher Scientific) and *MluI* (Thermo Fisher Scientific). Thereafter, the cassette was ligated into the pMSCV-PGK-Neo-IRES-GFP vector.

### Transfection

For retroviral production, the respective plasmid DNA was transfected into the packaging cell lines Plat-E (ecotropic) or Phoenix-GP (293GP, amphotropic) using turbofect transfectant reagent (Thermo Fisher Scientific, R0532). A mixture of 1000 μl of Opti-MEM I Reduced Serum Medium (Gibco, 31985-070), 10 μg of plasmid DNA and 20 μl turbofect transfectant reagent was incubated at RT for 20 min and added to fresh media on packaging cells drop-wise. For transfection of amphotropic Phoenix-GP cells, additionally 2 μg of the pCMV-VSV-G DNA were added for pseudotyping of the retroviruses. All reagent volumes were adjusted accordingly.

### Transduction

RAW264.7 cells were seeded at a density of 0.3 × 10^6^ cells/well in a 6 well-plate in 2 ml complete medium 12 h before transduction. RAW 264.7 is a macrophage cell line that was established from a tumor in a male mouse induced with the Abelson murine leukemia virus (ATCC). After removal of 1 ml full medium and addition of 2 ml of harvested retrovirus and 4 µg/ml polybrene (Sigma-Aldrich), cells were incubated for a period of 12 h at 37 °C under 5% CO_2_. Transduced RAW264.7 cells were selected with 8 μg/ml puromycin or 40 μg/ml blasticidin, until more than 95% of cells were GFP positive.

### Reporting summary

Further information on research design is available in the Nature Portfolio Reporting Summary linked to this article.

### Supplementary information

Supplementary Information

Reporting Summary

### Source data

Source Data file

## Data Availability

The microarray data have been deposited in GEO under accession code GSE229043. The mass spectrometry proteomics data have been deposited in the ProteomeXchange Consortium via the PRIDE^70^ partner repository under accession code PXD012036. Source data are provided with this paper.
