# Supplementary material for: ROCK1/2 signaling contributes to corticosteroid-refractory acute graft-versus-host disease
Source: Nat Commun. 2024 Jan 10;15:446. doi: 10.1038/s41467-024-44703-7 (PMC10781952; doi:10.1038/s41467-024-44703-7)
Supplement: Supplementary file 1 — Supplementary Information [file 41467_2024_44703_MOESM1_ESM.pdf]

ROCK1/2 signaling contributes to corticosteroid-refractory acute graft-versus-host disease

Kristina Maas-Bauer[1*], Anna-Verena Stell[1*], Kai-Li Yan[1*], Enrique de Vega[1,2*], Janaki Manoja Vinnakota[1*], Susanne Unger[3], Nicolas Núñez[3], Johana Norona[1], Nana Talvard-Balland[1], Stefanie Koßmann[1], Carsten Schwan[4], Cornelius Miething[1], Uta S. Martens[1,4], Khalid Shoumariyeh[1,5], Rosa P. Nestor[1], Sandra Duquesne[1], Kathrin Hanke[1], Michal Rackiewicz[6,7], Zehan Hu[6,7], Nadia El Khawanky[1], Sanaz Taromi[1], Hana Andrlova[1], Hemin Faraidun[2], Stefanie Walter[1], Dietmar Pfeifer[1], Marie Follo[1], Johannes Waldschmidt[1], Wolfgang Melchinger[1], Michael Rassner[1], Claudia Wehr[1], Annette Schmitt-Graeff[8], Sebastian Halbach[5,9], James Liao[10], Georg Häcker[11], Tilman Brummer[5,9,12], Joern Dengjel[6,7], Geoffroy Andrieux [13], Robert Grosse[4,14], Sonia Tugues[3], Bruce R Blazar[15], Burkhard Becher[3*], Melanie Boerries [5,13*], Robert Zeiser[1,5,12*]

[1] Department of Medicine I, Medical Center - University of Freiburg, Faculty of Medicine, University of Freiburg, Freiburg, Germany
[2] Faculty of Biology, University of Freiburg, Freiburg, Germany
[3] Institute of Experimental Immunology, University of Zurich, Zurich, Switzerland
[4] Institute of Experimental and Clinical Pharmacology and Toxicology, Medical Faculty, University of Freiburg, Freiburg, Germany
[5] German Cancer Consortium (DKTK), Partner Site Freiburg, a partnership between German Cancer Research Center (DKFZ) and Medical Center - University of Freiburg
[6] Department of Biology, University of Fribourg, Switzerland
[7] Department of Dermatology, Medical Center, University of Freiburg, Freiburg, Germany
[8] Institute of Pathology, University Hospital Freiburg, Freiburg, Germany
[9] IMMZ, University of Freiburg, Faculty of Medicine, Freiburg, Germany
[10] Department of Medicine, Section of Cardiology, University of Chicago, USA
[11] IMMH, University Hospital Freiburg, Faculty of Medicine, Freiburg, Germany
[12] Signaling Research Centres BIOSS and CIBSS - Centre for Integrative Biological Signaling Studies, University of Freiburg
[13] Institute of Medical Bioinformatics and Systems Medicine, Medical Center - University of Freiburg, Faculty of Medicine, University of Freiburg, Freiburg, Germany
[14] CIBSS-Centre for Integrative Biological Signalling Studies, University of Freiburg, Freiburg, Germany
[15] Department of Pediatrics, Division of Blood & Marrow Transplant & Cellular Therapy, University of Minnesota, Minneapolis, Minnesota, USA

* K.M.B, A.V.S., K.Y, E.V. J.M.V: These authors contributed equally,
* B.B., M.B. ,R.Z.: These authors jointly supervised this work

Table of contents

Supplemental Figure 1 1
Supplemental Figure 2 3
Supplemental Table 1 4
Supplemental Table 2 5
Supplemental Table 3 6
Supplemental Table 4 7
Supplemental Table 5 8
Supplemental Table 6 8
Supplemental Table 7 8

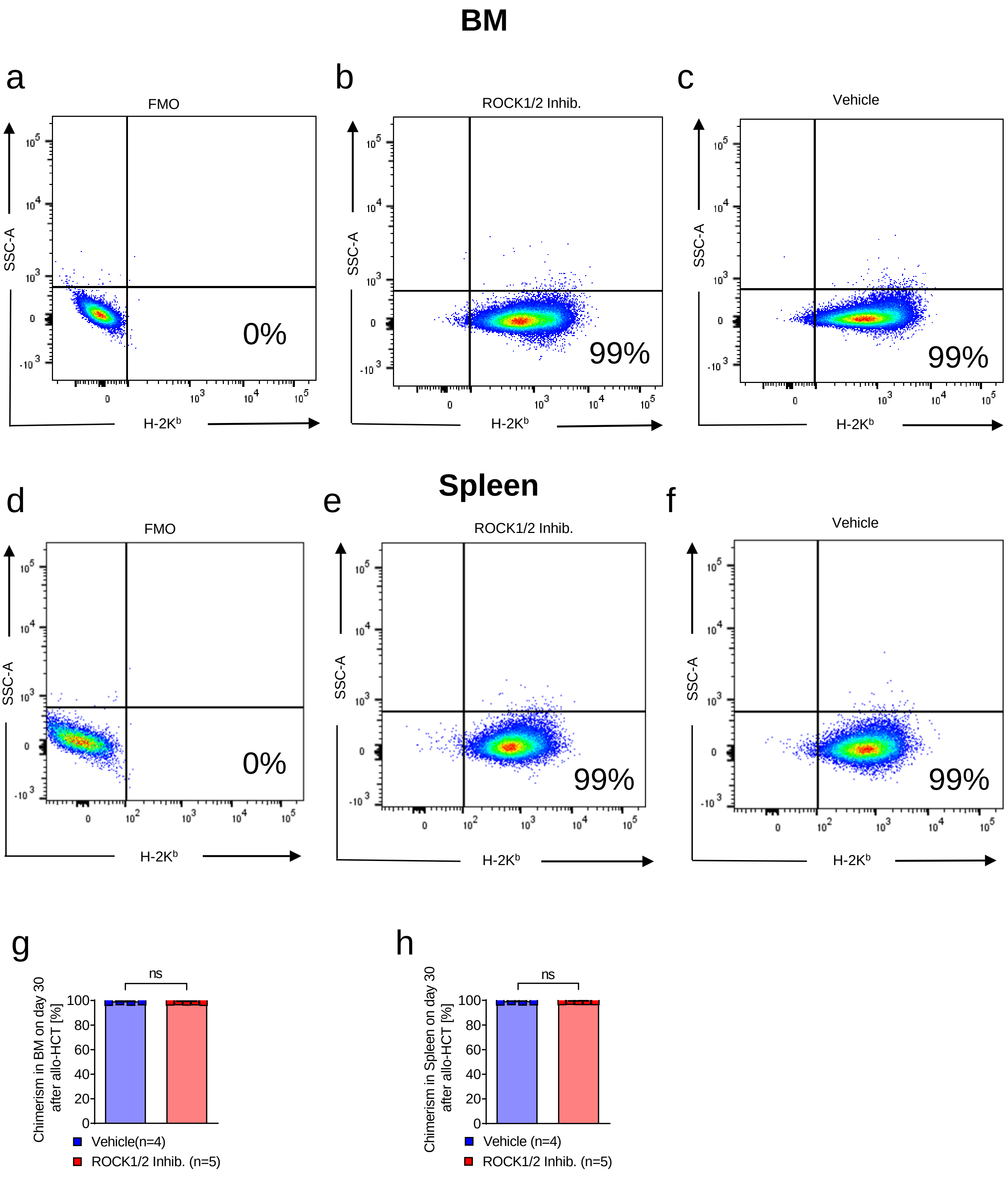
BM
a
FMO
SSC-A
H-2Kb
0%
b
ROCK1/2 Inhib.
SSC-A
H-2Kb
99%
c
Vehicle
SSC-A
H-2Kb
99%
Spleen
d
FMO
SSC-A
H-2Kb
0%
e
ROCK1/2 Inhib.
SSC-A
H-2Kb
99%
f
Vehicle
SSC-A
H-2Kb
99%
g
Chimerism in BM on day 30 after allo-HCT [%]
ns
Vehicle(n=4)
ROCK1/2 Inhib. (n=5)
h
Chimerism in Spleen on day 30 after allo-HCT [%]
ns
Vehicle (n=4)
ROCK1/2 Inhib. (n=5)

Suppl. Figure 1: ROCK1/2-inhibitor does not affect the engraftment after allo-HCT

a-h. Representative flow cytometry based chimerism analysis following bone marrow (BM) and T cell transplantation (donor C57BL/6 [H-2k^b] in recipient BALB/c [H-2k^d]). Mice in the ROCK1/2-inhibitor group were treated intraperitoneal with 8 mg/kg ROCK1/2-inhibitor (dissolved in 100 µl PBS) from day 4 to day 13, mice in the vehicle group were treated intraperitoneal with an equal volume PBS. Donor chimerism in BM cells and splenocytes was determined 30 days after transplantation. The recipient BM cells and splenocytes were stained with fluorescent monoclonal antibody to PerCP Cy5.5-conjucated H-2k^d and APC-conjucated H-2k^b. SSC-A, side scatter area. The experiment was performed once with n=5 in the ROCK1/2-inhibitor group and with n=4 in the vehicle group.

a-f. (a) negative control of H-2k^b in BM cells. (b) bone marrow chimerism in a recipient receiving ROCK1/2-inhibitor or (c) PBS (vehicle). (d) negative control of H-2k^b in splenocytes. (e) splenocytes chimerism in ROCK1/2-inhibitor group or (f) control group vehicle.

g-h. representative flow cytometric scatter plot of H-2k^b expression in bone marrow cells (g) and splenocytes (h) on day 30 after allo-HCT. Each point represents a mouse and *P*-value was calculated using an unpaired student t-test.

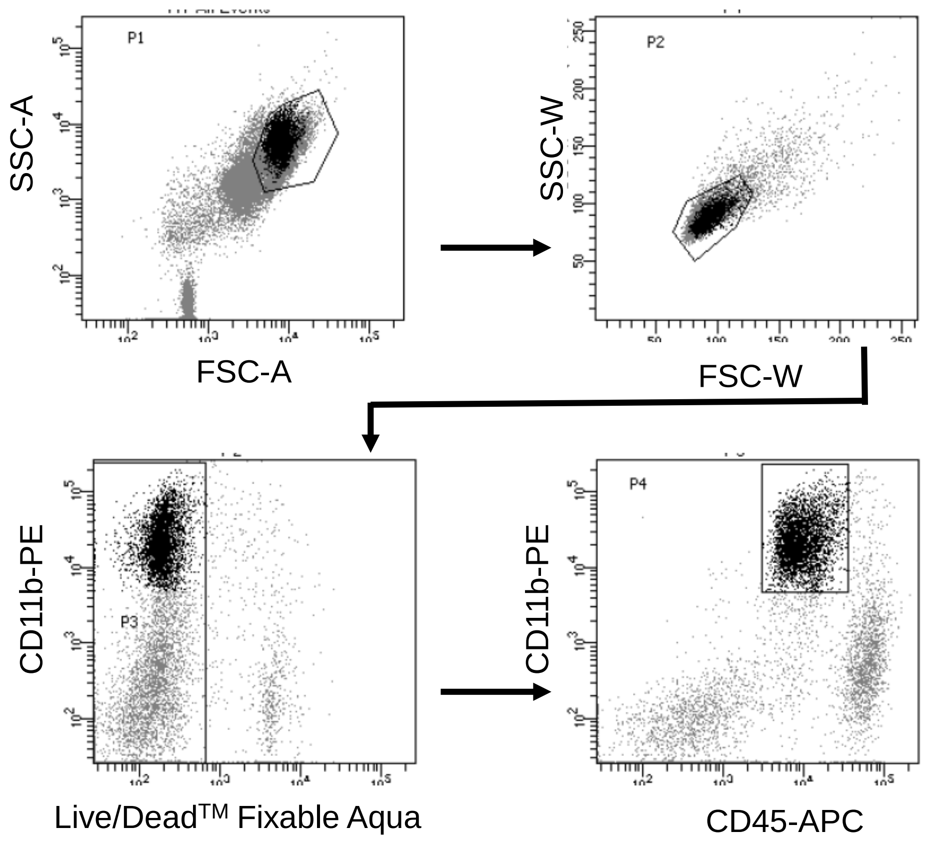

Suppl. Figure 2: Sorting Strategy for CD11b⁺ cells used in Figure 3f, 3g, 5a, 5b.

	Steroid-Responsive	Steroid-Refractory
Patient Characteristics		
Total number of Patients	7	12
Pt Age in Years (Median (Range)	63 (35-74)	61,5 (27-74)
Sex		
female	28,5 % (2)	41,7 % (5)
male	71,5 % (5)	58,3 % (7)
Primary Diagnosis		
AML	71,5 % (5)	41,7 % (5)
ALL	14,3 % (1)	16,7 % (2)
Lymphoma	14,3 % (1)	25 % (3)
Primary Myelofibrosis	-	8,3 % (1)
Chronic Neutrophil Leukemia	-	8,3 % (1)
Conditioning Regimen	100 % (7)	100 % (12)
MAC	-	-
RIC		
Donor Type	-	25 % (3)
MRD	-	-
MMRD	100 % (7)	25 % (3)
MUD	-	50 % (6)
MMUD		
Graft Type		
PBSC	100 % (7)	100 % (12)
GVHD Manifestation		
Skin	57 % (4)	83,3 % (10)
Intestine	100 % (7)	100 % (12)
Liver	-	8,3 % (1)
Eye	-	8,3 % (1)
Immunosuppression		
CyA	85,7 % (6)	83,3 % (10)
MMF	100 % (7)	91,7 % (11)
ATG	85,7 % (6)	50 % (6)
Cyclophosphamide	-	8,3 % (1)
Decortin	16,7 % (1)	100 % (12)
Ruxolitinib	-	25 % (3)
Everolimus	14,3 % (1)	16,7 % (2)
MPA	-	8,3 % (1)

Suppl. Table 1: Patient Characteristics – Intestinal Biopsies for the stainings for CD3 and CD14

Total number of patients	6
Variable	
	median (range)
Pt. age in years	49 (28-74)
Gender	% (absolute number)
female	66.7 (4)
male	33.3 (2)
Primary Diagnosis	
AML	50.0 (3)
ALL	33.3 (2)
Lymphoma	16.7 (1)
Conditioning Regimen	
MAC	100 (6)
RIC	0 (0)
Donor Type	
MRD	16.7 (1)
MUD	50 (3)
MMUD	33.3 (2)
Graft Source	
PBSC	100 (6)
GvHD Manifestation	
Skin	100 (6)
Intestines	50.0 (3)
Liver	16.7 (1)
Immunosuppression	
CyA	100 (6)
MMF	100 (6)
ATG	83.3 (5)
Prednisone	83.3 (5)
Cyclophosphamide	16.7 (1)

ECP	16.7 (1)
Ruxolitinib	33.3 (2)

Abbreviations: AML: acute myeloid leukemia, ALL: acute lymphoid leukemia, MAC: Myeloablative conditioning, RIC: reduced intensity conditioning, MRD: matched related donor, MUD: matched unrelated donor, MMUD: mismatched unrelated donor, PBSC: peripheral blood stem cells, CyA: cyclosporine A, MMF: mycophenolate mofetil, ATG: Anti Thymocyte globulin (Grafalon®), immunosuppression (CyA, MMF and ATG were used as prophylaxis, Decortin, ECP and ruxolitinib were used to treat GVHD.

Suppl. Table 2: SR-GVHD Patient Group analyzed via mass spectrometry – Patients characteristics; Chemotherapy conditioning regimens; transplant characteristics; GvHD manifestation and immunosuppression

Total number of patients<br><br>Variable	6
	median (range)
Pt. age in years	63 (28-71)
Sex	% (absolute number)
female	50.0 (3)
male	50.0 (3)
Primary Diagnosis	
AML	66.7 (4)
MM	33.3 (2)
Conditioning Regimen	
MAC	83.3 (5)
RIC	16.7 (1)
Donor Type	
MMRD	16.7 (1)
MUD	33.3 (2)
MMUD	50.0 (3)
Graft Source	
PBSC	100 (6)
Previous GvHD Manifestation	
No GvHD	50.0 (3)

Skin	50.0 (3)
<u>Immunosuppression</u>	
CyA	100 (6)
MMF	100 (6)
ATG	83.3 (5)
Cyclophosphamide	16.7 (1)

Abbreviations: AML: acute myeloid leukemia, MM: multiple myeloma, MAC: Myeloablative conditioning, RIC: reduced intensity conditioning, MRD: matched related donor, MUD: matched unrelated donor, MMUD: mismatched unrelated donor, PBSC: peripheral blood stem cells, CyA: cyclosporine A, MMF: mycophenolate mofetil, ATG: Anti Thymocyte globulin

Suppl. Table 3: Steroid-responsive Patient Group analyzed via mass spectrometry – Patients characteristics; Chemotherapy conditioning regimens; transplant characteristics; previous GvHD manifestation; immunosuppression at time point of blood collection

FACS	Antibody	Clone	Dilution	Cat.#	Supplier
	anti-CD45	30-F11	1:200	103132	Biolegend
	anti-CD45.1	A20	1:200	110736	Biolegend
	anti-CD45.2	104	1:200	109808	Biolegend
	anti-CD11b	M1/70	1:200	101224	Biolegend
	anti-Ly6G	1A8	1:200	127626	Biolegend
	anti-CD11c	N418	1:200	117322	Biolegend
	anti-I-Ab	AF6-120.1	1:200	116418	Biolegend
	anti-I-A/E	M5/114.15.2	1:200	25-5321-82	Invitrogen
	anti-CD80	15-10A1	1:100	104713	Biolegend
	anti-CD86	GL-1	1:100	105005	Biolegend
	anti-CD3	17A2	1:200	100213	BD Bioscience
	anti-CD4	GK1.5	1:200	100431	Biolegend
	anti-CD8a	53-6.7	1:100	100712	Biolegend
	anti-H2k^{b}	AF6-88.5	1:200	116518	Biolegend
	anti-H2k^{d}	SF1-1.1	1:200	116618	Biolegend
	MHC Class II (I-A/I-E)	M5/114.15.2	1:100	17-5323-82	Invitrogen
	Granzyme B	NGZB	1:100	25-8898-82	Invitrogen
	Perforin	S16009A	1:100	154304	Biolegend
	CellTrace^{TM} violet		5mM	C34557	Invitrogen
	anti-CD19	6D5	1:400	115520	Biolegend
	Zombie NIR fixable viability dye		1:500	423106	Biolegend
	Live/Dead^{TM} Fixable Aqua Dead Cell Stain Kit		1:500	L34957	Invitrogen
**Microscopy**	Phalloidin Alexa Fluor 488^{TM}	M1/70	1:100	A12379	Thermo Fisher scientific

Suppl. Table 4: Antibodies for FACS and microscopy

Antibodies	Concentration	Cat. #	Company
ROCK1 (C8F7) anti-rabbit	1:1000	4035S	Cell signaling Technology
NF-kB p65 (D14E12) XP ®	1:2000	8242S	Cell signaling Technology
pNF-kB p65 (Ser536) (93H1)	1:2000	3033S	Cell signaling Technology
Cofilin (D59) anti-rabbit	1:1000	3318S	Cell signaling Technology
pCofilin (Ser3) (77G2) anti-rabbit	1:1000	3313S	Cell signaling Technology
Vinculin (E1E9V) XP® anti-rabbit	1:2000	13901	Cell signaling Technology
β-actin (13E5) anti-rabbit	1:2000	4970	Cell signaling Technology
HRP-linked anti-rabbit IgG	1:5000	#7074	Cell signaling Technology
The PageRuler™ Prestained Protein ladder	10-180 kDa	22616	Thermo Fisher Scientific

Suppl. Table 5: Antibodies for western blots

Target	Direction	Sequence 5'—3'
mGAPDH	fwd	TTC ACC ACC ATG GAG AAG GC
	rev	GGC ATG GAC TGT GGT CAT GA
mHPRT	fwd	AGC CTAA AGA TGA GCG CAA GT
	rev	TTA CTA GGC AGA TGG CCA CA
mIL-6	fwd	CTC TGC AAG AGA CTT CCA TCCA
	rev	GAC AGG TCT GTT GGG AGT GG
mIl-10	fwd	CGG GAA GAC AAT AAC TGC ACCC
	rev	CGG TAA GCA GTA TGT TGT CCA GC
miNOS	fwd	GAG ACA GGG AAG TCT GAA GCAC
	rev	CCA GCA GTA GTT GCT CCT CTTC
mTnfa	fwd	CCA CCA TCA AGG ACT CAA
	rev	ATC TTA TCC AGC CTC ATT CT
mIl-1β	fwd	GCA ACT GTT CCT GAA CTC AACT
	rev	ATC TTT TGG GGT CCG TCA ACT

The primers for qPCR were self-designed and commercially synthesized by Eurofins Genomics or Integrated DNA Technologies Germany GmbH.

Suppl. Table 6: qRt-PCR primers

sh*ROCK1*.5706	5'-TGCTGTTGACAGTGAGCGCGCATAATGAAATGCAATTAAATAG TGAAGCCACAGATGTATTTAATTGCATTTCATTATGCATGCCTACT GCCTCGGA-3´
sh*ROCK1*.1791	5'-TGCTGTTGACAGTGAGCGCGCATAATGAAATGCAATTAAATAG TGAAGCCACAGATGTATTTAATTGCATTTCATTATGCATGCCTACT GCCTCGGA-3'
sh*Renilla* luciferase	5'-TGCTGTTGACAGTGAGCGCAGGAATTATAATGCTTATCTATAG TGAAGCCACAGATGTATAGATAAGCATTATAATTCCTATGCCTACT GCCTCGGA-3'

Suppl. Table 7: ShRNA oligonucleotide sequences
