# Supplementary material for: ROCK1/2 signaling contributes to corticosteroid-refractory acute graft-versus-host disease
Source: Nat Commun. 2024 Jan 10;15:446. doi: 10.1038/s41467-024-44703-7 (PMC10781952; doi:10.1038/s41467-024-44703-7)
Supplement: Supplementary file 2 — Reporting Summary [file 41467_2024_44703_MOESM2_ESM.pdf]

nature portfolio

Corresponding author(s): Robert Zeiser
Last updated by author(s): Dec 5, 2023

Reporting Summary

Nature Portfolio wishes to improve the reproducibility of the work that we publish. This form provides structure for consistency and transparency in reporting. For further information on Nature Portfolio policies, see our Editorial Policies and the Editorial Policy Checklist.

Statistics

For all statistical analyses, confirm that the following items are present in the figure legend, table legend, main text, or Methods section.

n/a	Confirmed	
☐	☒	The exact sample size (*n*) for each experimental group/condition, given as a discrete number and unit of measurement
☐	☒	A statement on whether measurements were taken from distinct samples or whether the same sample was measured repeatedly
☐	☒	The statistical test(s) used AND whether they are one- or two-sided<br>*Only common tests should be described solely by name; describe more complex techniques in the Methods section.*
☒	☐	A description of all covariates tested
☐	☒	A description of any assumptions or corrections, such as tests of normality and adjustment for multiple comparisons
☐	☒	A full description of the statistical parameters including central tendency (e.g. means) or other basic estimates (e.g. regression coefficient) AND variation (e.g. standard deviation) or associated estimates of uncertainty (e.g. confidence intervals)
☐	☒	For null hypothesis testing, the test statistic (e.g. *F*, *t*, *r*) with confidence intervals, effect sizes, degrees of freedom and *P* value noted<br>*Give P values as exact values whenever suitable.*
☒	☐	For Bayesian analysis, information on the choice of priors and Markov chain Monte Carlo settings
☒	☐	For hierarchical and complex designs, identification of the appropriate level for tests and full reporting of outcomes
☒	☐	Estimates of effect sizes (e.g. Cohen's *d*, Pearson's *r*), indicating how they were calculated

Our web collection on statistics for biologists contains articles on many of the points above.

Software and code

Policy information about availability of computer code

Data collection	Not applicable
Data analysis	Proteomics analysis: For data analysis the Perseus computational platform was used. Briefly, quantified proteins were filtered for kinases and only kinases identified in all 14 samples (6 samples per group plus one technical replicate) were further considered. Log2 transformed iBAQ values were used to identify significantly enriched kinases comparing GVHD to non-GVHD samples using a two-sided t-test, FDR 0.05, S0=0.1.<br>Western blots: Quantification of Western blots was done using LabImage 1D INTAS software.<br>Microarray: Microarray analysis was performed using Affymetrix Clariom S Mouse arrays. Arrays were normalized using Robust Multi-Array Average expression measure32, and probe expression values summarized to the gene level using the R/Bioconductor package pd.clariom.s.mouse. Differential analysis: A linear model based approach (limma R package)33 was used to identify the differentially regulated RNA between untreated and treated cells. Regulated RNA with Benjamini Hochberg adjusted P-value <0.05 was set as significant.<br>For statistical analysis, an unpaired or paired t-test (two-sided) or when required a Two-way ANOVA test was applied. If the data did not meet the criteria for normality, the Mann-Whitney U test was applied unless stated otherwise in the Figure legend. Data are presented as mean and standard deviation (error bars). Differences were considered significant when the P-value was <0.05. Survival data were plotted using the Kaplan-Meier method and compared by using a Log-rank (Mantel-Cox) test. Statistical analysis was performed using GraphPad Prism (GraphPad Software V9,; San Diego, CA). Data are presented as mean and s.e.m. (error bars). Differences were considered significant when the P-value was <0.05.

For manuscripts utilizing custom algorithms or software that are central to the research but not yet described in published literature, software must be made available to editors and reviewers. We strongly encourage code deposition in a community repository (e.g. GitHub). See the Nature Portfolio guidelines for submitting code & software for further information.

Data

Policy information about availability of data

All manuscripts must include a data availability statement. This statement should provide the following information, where applicable:

- Accession codes, unique identifiers, or web links for publicly available datasets
- A description of any restrictions on data availability
- For clinical datasets or third party data, please ensure that the statement adheres to our policy

Data Availability
Source data are provided with this paper.
The microarray data have been deposited in GEO under accession code GSE229043:
Got to https://www.ncbi.nlm.nih.gov/geo/query/acc.cgi?acc=GSE229043; Enter token cnevemmsfzsnzgp into the box.
The mass spectrometry proteomics data have been deposited to the ProteomeXchange Consortium via the PRIDE77 partner repository with the dataset identifier PXD012036.
Project Name: ROCK1 inhibition reduces steroid-refractory acute graft-versus-host disease
Project accession: PXD012036, Reviewer account details:
Username: reviewer50925@ebi.ac.uk, Password: RTrmaj5A"

Code Availability
Microarray: GEO under accession code GSE229043:
Got to https://www.ncbi.nlm.nih.gov/geo/query/acc.cgi?acc=GSE229043; Enter token cnevemmsfzsnzgp into the box.
The mass spectrometry proteomics: ProteomeXchange Consortium dataset identifier PXD012036
Project accession: PXD012036, Reviewer account details:
Username: reviewer50925@ebi.ac.uk, Password: RTrmaj5A"

Research involving human participants, their data, or biological material

Policy information about studies with human participants or human data. See also policy information about sex, gender (identity/presentation), and sexual orientation and race, ethnicity and racism.

Reporting on sex and gender	We reported the sex of the patients in our population based on self-reporting. We do not provide information on sex on the individual patient level but only summarize the number of female and male patients. Informed consent for sharing individual level data was obtained prior to the study.
Reporting on race, ethnicity, or other socially relevant groupings	N/A
Population characteristics	Patients who underwent allo-HCT at the University hospital Freiburg were included in the study. Patients with aGVHD were defined as non-responsive to corticosteroids (SR-aGVHD) if they had received prednisolone at a dose of 2 mg/kg bodyweight for at least 7 days without response or when aGVHD progression was documented after five days of treatment and the clinical situation required additional immunosuppressive therapy. As indicated, the control groups consisted (a) of patients who had aGVHD responsive to corticosteroids and achieved at least a partial remission to steroids, (b) patients post allo-HCT who never developed aGVHD or (c) healthy controls.<br>Population for intestinal biopsies (shown in Figure 1a und b(left and middle panel):<br>Characteristics SR-aGVHD: Age in years: 27-74 (median: 61,5), Gender: 5 female, 7 male, diagnosis (n): AML (5), ALL (2), lymphoma (3), myelofibrosis (1), chronic neutrophil leukemia (1)<br>Characteristics steroid responsive GVHD: Age in years: 35-74 (median: 63, Gender: 2 female, 5 male, diagnosis (n): AML (5), ALL (1), lymphoma (1)<br>Population for mass spectrometry (shown in Figure 2a):<br>SR-aGVHD: 6 Patients, age 28-74 (median 49), gender: 4 female, 4 male, diagnosis (n): AML (3), ALL (2), lymphoma (1), conditioning (n): MAC (6), graft source (n): PBSC (6), GVHD manifestation (n): skin (6,), intestinea (3), liver (1)<br>steroid responsive aGVHD: 6 Patients, age 28-71 (median 63), gender: 3 female, 3 male, diagnosis (n): AML (4), MM (2), conditioning (n): MAC (5), RIC (1), graft source (n): PBSC (6), GVHD manifestation (n): no (3) skin (3,)
Recruitment	All patients who underwent allo-HCT at the University hospital Freiburg and fulfilled the inclusion criteria during the study period were included in the study. No patients were excluded. Patients with aGVHD were defined as non-responsive to corticosteroids (SR-aGVHD) if they had received prednisolone at a dose of 2 mg/kg bodyweight for at least 7 days without response or when aGVHD progression was documented after five days of treatment and the clinical situation required additional immunosuppressive therapy. As indicated, the control groups consisted (a) of patients who had aGVHD responsive to corticosteroids and achieved at least a partial remission to steroids, (b) patients post allo-HCT who never developed aGVHD or (c) healthy controls.
Ethics oversight	Human sample collection and analysis were approved by the Institutional Ethics Review Board of the Medical center, University of Freiburg, Germany). Written informed consent was obtained from each patient. All analysis of human data was carried out in compliance with relevant ethical regulations.<br>The study was approved by the local ethics committee (project-number: 20-1223). TSPO- PET images of 22 age-matched healthy controls were obtained from previous cohorts (64, 65), approved by the local ethics committee (project-numbers:

17-569, 19-022). Written informed consent was obtained from all participants in accordance with the Declaration of Helsinki.

Note that full information on the approval of the study protocol must also be provided in the manuscript.

Field-specific reporting

Please select the one below that is the best fit for your research. If you are not sure, read the appropriate sections before making your selection.

- [x] Life sciences
- [ ] Behavioural & social sciences
- [ ] Ecological, evolutionary & environmental sciences

For a reference copy of the document with all sections, see nature.com/documents/nr-reporting-summary-flat.pdf

Life sciences study design

All studies must disclose on these points even when the disclosure is negative.

Sample size

For sample size in the murine survival experiments a power analysis was performed. A sample size of at least n=8 per group was determined by 80% power to reach a statistical significance of 0.05 in order to detect an effect size of at least 1.06.
All other experiments were performed with 4-10 biological individuals, usually in 2-3 independent experiments. The sample size was chosen based on typical sample sizes in GVHD experiments as previously reported e.g. Schwab et al., Nat. Med. 2014.

Data exclusions

No data were excluded from the analyses.

Replication

All the experimental findings were reproduced with 2 or 3 independent experiments (except supplemental Figure 1 which was performed only once with 4-5 biological individuals in each group).

Randomization

There was no randomization of mice or samples before analysis. Therefore, all samples or mice were included in our analysis.

Blinding

The experiments were performed in a non-blinded manner, because the survival is not a subjective parameter. The histological scoring was done in a blinded fashion by a pathologist not aware of the treatment groups. This approach is commonly used in GVHD experiments (e.g. Schwab et al., Nat. Med. 2014, Wilhelm et al., Nat. Med. 2010).

Reporting for specific materials, systems and methods

We require information from authors about some types of materials, experimental systems and methods used in many studies. Here, indicate whether each material, system or method listed is relevant to your study. If you are not sure if a list item applies to your research, read the appropriate section before selecting a response.

Materials & experimental systems

n/a	Involved in the study	
☐	☒	Antibodies
☐	☒	Eukaryotic cell lines
☒	☐	Palaeontology and archaeology
☐	☒	Animals and other organisms
☒	☐	Clinical data
☒	☐	Dual use research of concern
☒	☐	Plants

Methods

n/a	Involved in the study	
☒	☐	ChIP-seq
☐	☒	Flow cytometry
☒	☐	MRI-based neuroimaging

Antibodies

Antibodies used

FACS Antibody Clone Dilution Cat.# Supplier
anti-CD45 30-F11 1:200 103132 Biolegend
anti-CD45.1 A20 1:200 110736 Biolegend
anti-CD45.2 104 1:200 109808 Biolegend
anti-CD11b M1/70 1:200 101224 Biolegend
anti-Ly6G 1A8 1:200 127626 Biolegend
anti-CD11c N418 1:200 117322 Biolegend
anti-I-Ab AF6-120.1 1:200 116418 Biolegend
anti-I-A/E M5/114.15.2 1:200 25-5321-82 Invitrogen
anti-CD80 15-10A1 1:100 104713 Biolegend
anti-CD86 GL-1 1:100 105005 Biolegend
anti-CD3 17A2 1:200 100213 BD Bioscience
anti-CD4 GK1.5 1:200 100431 Biolegend
anti-CD8a 53-6.7 1:100 100712 Biolegend

anti-H2kb AF6-88.5 1:200 116518 Biolegend
anti-H2kd SF1-1.1 1:200 116618 Biolegend
MHC Class II (I-A/I-E) M5/114.15.2 1:100 17-5323-82 Invitrogen
Granzyme B NGZB 1:100 25-8898-82 Invitrogen
Perforin S16009A 1:100 154304 Biolegend
CellTraceTM violet 5mM C34557 Invitrogen
anti-CD19 6D5 1:400 115520 Biolegend
Zombie NIR fixable viability dye 1:500 423106 Biolegend
Microscopy Phalloidin Alexa Fluor 488TM M1/70 1:100 A12379 Thermo Fisher scientific
AAntibodies Concentration Cat. # Company
ROCK1 (C8F7) anti-rabbit 1:1000 4035S Cell signaling Technology
NF-kB p65 (D14E12) XP ® 1:2000 8242S Cell signaling Technology
pNF-kB p65 (Ser536) (93H1) 1:2000 3033S Cell signaling Technology
Cofilin (D59) anti-rabbit 1:1000 3318S Cell signaling Technology
pCofilin (Ser3) (77G2) anti-rabbit 1:1000 3313S Cell signaling Technology
Vinculin (E1E9V) XP® anti-rabbit 1:2000 13901 Cell signaling Technology
β-actin (13E5) anti-rabbit 1:2000 4970 Cell signaling Technology
HRP-linked anti-rabbit IgG 1:5000 #7074 Cell signaling Technology
The PageRuler™ Prestained Protein ladder 10-180 kDa 22616 Thermo Fisher Scientific
Suppl. Table 5: Antibodies for western blots

Validation

Antibodies were validated by titration experiments where the base line was the recommended working concentration provided by the manufacturer. Validation was performed per experimental setup and used cell type.
The dosage for the ROCK1/2 inhibitor was based on previous publications (Tomomasa T et al., Life Sci. 2000, Meng et al., Mol Oncol. 2020)

Eukaryotic cell lines

Policy information about cell lines and Sex and Gender in Research

Cell line source(s)

Mouse cell lines:

MLL-PTD/wt FLT3-ITD/wt AML cells are derived from an in vivo AML model and not a cell line that can be expanded in vitro (Matthew N et al. Nat Med 2018)
WEHI-3B (ATCC cell line)
RAW264.7 cells (ATCC cell line)

Authentication

The cell lines used in this study were obtained from a batch that had been prevoiusly authenticated at DSMZ, Germany.
WEHI-3B (ATCC cell line): bought freshly from DKFZ 2022
RAW264.7 cells (ATCC cell line) : STR profile done by the company Microsynth AG (Basel, Switzerland)

Mycoplasma contamination

The cell lines used in this study were obtained from a batch that had been prevoiusly tested for Mycoplasma contamination (PCR analysis) and were found to be negative. Cell lines were subsequently routinely tested for mycoplasma contamination.

Commonly misidentified lines
(See ICLAC register)

N/A

Animals and other research organisms

Policy information about studies involving animals; ARRIVE guidelines recommended for reporting animal research, and Sex and Gender in Research

Laboratory animals

C57BL/6 (H-2Kb) and BALB/c (H-2Kd) mice were purchased from Janvier Labs (France) or from the local stock of the animal facility at University of Freiburg. Mice were used between 6 and 14 weeks of age, and only female or male donor/recipient pairs were used. Housing conditions: dark/light 12/12 hours, temperature: 20-24°C, humidity 45-65%

Wild animals

The study did not involve wild animals.

Reporting on sex

To rule out sex specific differences we used female donors and female recipients or male donors and male recipients.

Field-collected samples

The study did not involve samples collected from the field.

Ethics oversight

All mouse experiments were approved by the Federal Ministry for Nature, Environment and Consumers' Protection of the state of Baden-Württemberg (Regierungspräsidium Freiburg, Freiburg, Germany) (No: G-22/103, G17/63, G18/36, G20/078, G22/007).

Note that full information on the approval of the study protocol must also be provided in the manuscript.

Plants

Seed stocks

Report on the source of all seed stocks or other plant material used. If applicable, state the seed stock centre and catalogue number. If plant specimens were collected from the field, describe the collection location, date and sampling procedures.

Novel plant genotypes

Describe the methods by which all novel plant genotypes were produced. This includes those generated by transgenic approaches, gene editing, chemical/radiation-based mutagenesis and hybridization. For transgenic lines, describe the transformation method, the number of independent lines analyzed and the generation upon which experiments were performed. For gene-edited lines, describe the editor used, the endogenous sequence targeted for editing, the targeting guide RNA sequence (if applicable) and how the editor was applied.

Authentication

Describe any authentication procedures for each seed stock used or novel genotype generated. Describe any experiments used to assess the effect of a mutation and, where applicable, how potential secondary effects (e.g. second site T-DNA insertions, mosiacism, off-target gene editing) were examined.

Flow Cytometry

Plots

Confirm that:

☒ The axis labels state the marker and fluorochrome used (e.g. CD4-FITC).

☒ The axis scales are clearly visible. Include numbers along axes only for bottom left plot of group (a 'group' is an analysis of identical markers).

☒ All plots are contour plots with outliers or pseudocolor plots.

☒ A numerical value for number of cells or percentage (with statistics) is provided.

Methodology

Sample preparation

BM transplantation experiments were performed as described. The major mismatch strain combinations used were C57BL/6 into BALB/c or BALB/c into C57BL/6 as indicated in the respective experiments. Briefly, recipients were injected by intravenous injection (i.v.) via tail vein with 5x106 BM cells after lethal irradiation with 1000 cGy (BALB/c recipient) or 1100 cGy (C57BL/6 recipient), using a 137Cs source split into two equal doses and four hours apart. To induce aGVHD, CD4+ and CD8+ T cells were isolated from donor spleens and enriched with the MACS cell separation system and the Pan T Cell Isolation Kit II (Miltenyi Biotec, Germany) according to the manufacturer's instructions. CD4+/CD8+ T cells were given at a dosage of 0.3-0.5x106 on day 0 as indicated in the respective experiments.

Instrument

The majority of data were acquired on a BD LSR Fortessa and BD Fusion for cell sorting.

Software

The majority of data were analyzed using FlowJo (Flowjo 10.4 or 10.6, LLC) software.

Cell population abundance

The gating strategy was determined using unstained controls, single stains and fluorescence minus one (FMO) controls.

Gating strategy

All experiments were performed using appropiated controls and compensation controls were also considered. Cells were first gated using SSC-A vs FSC-A, doublet cells were excluded by gating on FSC-H vs FSC-A and subsequent SSC-H vs SSC-A, dead cells were excluded using LIVE/DEAD™ Fixable Aqua Dead Cell Stain Kit (L34966) or Zombie Red™ Fixable Viability Kit (423109), Subsequently, the desired paramiters were gated. Unstained controls, single stains and fluorescence minos one (FMO) controls were used for appropiate gating on desired parameters and differentiation between positive and negative cells. Gating strategy will be provided upon request

☒ Tick this box to confirm that a figure exemplifying the gating strategy is provided in the Supplementary Information.
